# Blood-spinal cord barrier leakage is independent of motor neuron pathology in ALS

**DOI:** 10.1186/s40478-021-01244-0

**Published:** 2021-08-26

**Authors:** Sarah Waters, Molly E. V. Swanson, Birger V. Dieriks, Yibin B. Zhang, Natasha L. Grimsey, Helen C. Murray, Clinton Turner, Henry J. Waldvogel, Richard L. M. Faull, Jiyan An, Robert Bowser, Maurice A. Curtis, Mike Dragunow, Emma Scotter

**Affiliations:** 1 Department of Pharmacology and Clinical Pharmacology, University of Auckland, Auckland, New Zealand; 2 Department of Anatomy and Medical Imaging, University of Auckland, Auckland, New Zealand; 3 Centre for Brain Research, University of Auckland, Auckland, New Zealand; 4 LabPlus, Auckland City Hospital, Auckland, New Zealand; 5 Departments of Neurology and Neurobiology, Barrow Neurological Institute, Phoenix, ARI 85013 USA; 6 Present Address: School of Biological Sciences, University of Auckland, Auckland, New Zealand

**Keywords:** Blood–brain barrier, Blood-spinal cord barrier, TDP-43, ALS, Hemoglobin, Human

## Abstract

Amyotrophic lateral sclerosis (ALS) is a fatal neurodegenerative disease involving progressive degeneration of upper and lower motor neurons. The pattern of lower motor neuron loss along the spinal cord follows the pattern of deposition of phosphorylated TDP-43 aggregates. The blood-spinal cord barrier (BSCB) restricts entry into the spinal cord parenchyma of blood components that can promote motor neuron degeneration, but in ALS there is evidence for barrier breakdown. Here we sought to quantify BSCB breakdown along the spinal cord axis, to determine whether BSCB breakdown displays the same patterning as motor neuron loss and TDP-43 proteinopathy. Cerebrospinal fluid hemoglobin was measured in living ALS patients (n = 87 control, n = 236 ALS) as a potential biomarker of BSCB and blood–brain barrier leakage. Cervical, thoracic, and lumbar post-mortem spinal cord tissue (n = 5 control, n = 13 ALS) were then immunolabelled and semi-automated imaging and analysis performed to quantify hemoglobin leakage, lower motor neuron loss, and phosphorylated TDP-43 inclusion load. Hemoglobin leakage was observed along the whole ALS spinal cord axis and was most severe in the dorsal gray and white matter in the thoracic spinal cord. In contrast, motor neuron loss and TDP-43 proteinopathy were seen at all three levels of the ALS spinal cord, with most abundant TDP-43 deposition in the anterior gray matter of the cervical and lumbar cord. Our data show that leakage of the BSCB occurs during life, but at end-stage disease the regions with most severe BSCB damage are not those where TDP-43 accumulation is most abundant. This suggests BSCB leakage and TDP-43 pathology are independent pathologies in ALS.

**Supplementary Information:**

The online version contains supplementary material available at 10.1186/s40478-021-01244-0.

## Introduction

Amyotrophic lateral sclerosis (ALS) is characterized by the progressive and eventually fatal degeneration of upper and lower motor neurons [1–3]. Motor symptom onset is focal, beginning most commonly in the limbs, less commonly in the muscles of speech and swallowing, and rarely in the respiratory muscles [1–3]. Motor neurons that innervate the area of first symptom onset show the greatest cell loss and pathologic deposition of TDP-43 [4, 5]. A wealth of evidence shows that cell-autonomous TDP-43 dysfunction initiates motor neuron degeneration [6–8], and indeed phosphorylated TDP-43 protein inclusions are seen in almost all ALS cases [9, 10]. However, degeneration in ALS animal models can be modified by non-cell autonomous factors including glial activity and potentially also by the integrity of the blood–brain and blood-spinal cord barriers [8, 11].

The blood–brain barrier (BBB), blood-spinal cord barrier (BSCB), and blood-cerebrospinal fluid (blood-CSF) barrier are specialized interfaces that regulate the influx of nutrients and ions into the central nervous system, and the removal of waste and other solutes [12, 13]. In addition, these barriers separate the brain, spinal cord parenchyma, and CSF respectively from potentially neurotoxic blood-borne components in the circulation, such as peripheral leukocytes, erythrocytes and plasma proteins [14–16]. In ALS, the BBB and BSCB are compromised (literature from human studies summarized in Additional file 1: Table S1). Their disruption is evidenced by extravasation of blood components such as immunoglobulin G (IgG), fibrin, thrombin and the erythrocyte components hemoglobin and hemosiderin into the brain and spinal cord parenchyma [17–20]. Blood protein concentrations are also altered in ALS CSF [21], implicating either blood-CSF barrier disruption [22], and/or diffusion of BBB and BSCB leakage factors from the brain and spinal cord interstitial fluid into the CSF. In mice, many of the blood components that leak across the BBB, BSCB, or blood-CSF barrier act as neurotoxins [23–27] which, when allowed into contact with motor neurons, are likely to enhance motor neuron vulnerability to autonomous TDP-43-mediated damage. Yet it remains unclear whether brain barrier leakage in ALS causes, is caused by, or is independent from motor neuron damage.

Causation or correlation could be revealed by the patterning of barrier leakage relative to motor neuron pathology. Motor neuron cell bodies in the spinal cord are situated in the anterior horns of the gray matter, and motor neuron degeneration in ALS is most severe at the cervical and lumbar levels of the cord [5, 28]. Because of this, breakdown of the BSCB and leakage of blood components into the spinal cord parenchyma has been studied almost exclusively in the cervical and lumbar anterior horns, with little consideration given to areas with less severe motor neuron pathology. Endothelial and pericyte degeneration [17, 29] and detachment of astrocyte endfeet [30] were examined only in the cervical or lumbar, or unspecified, anterior horns. Reduced expression of tight junction mRNA and proteins have been found in cervical and/or lumbar cord homogenates [18, 31], but the anatomical relationship with preserved or degenerating motor neurons was lost. And striking leakage of plasma IgG, fibrin, thrombin, and hemoglobin occurs into the cervical anterior horn parenchyma [17], all of which are neurotoxic in mice [23–27], but the severity of leakage was not examined elsewhere. These studies therefore suggest a relationship between motor neuron and BSCB pathologies, but the two have never been mapped in parallel and in detail across multiple levels of the spinal cord to examine their correlation.

Motor neuron damage and death in most cases of ALS is initiated by aggregation, mislocalization and loss of RNA processing function of TDP-43 [6, 32]. Thus, the regional distribution of neuronal TDP-43 protein inclusions across the brain and spinal cord is positively correlated with the regions of greatest neuronal death [4, 5, 33]. In the spinal cord, TDP-43 proteinopathy and neuronal loss are greatest at the cervical or lumbar enlargements, depending on the site of symptom onset; upper limb-onset cases show most severe pTDP-43 pathology and motor neuron loss at the cervical enlargement, while lower limb-onset pathology is most severe at the lumbar enlargement [5, 34]. Severe phospho-TDP-43 and motor neuron pathology can be further localized to the dorsolateral motor nuclei that innervate the distal limbs [5], with the distal limbs being first affected in the majority of non-bulbar ALS patients [35, 36]. This pattern of TDP-43 proteinopathy and motor neuron loss along the spinal cord axis provides a well-characterized setting against which to determine any correlation with BSCB compromise.

Here we quantify BSCB leakage, TDP-43 proteinopathy, and motor neuron loss, along the ALS spinal cord axis. We examine the spatial relationship between BSCB compromise and the development of proteinopathy and neurodegeneration to determine whether BSCB leakage may be a contributor to human ALS pathogenesis and thus a target for therapy.

## Materials and methods

### CSF samples

Cerebrospinal fluid samples provided by the NEALS Biofluid Repository, Boston, MA and Phoenix, AZ, were obtained via lumbar puncture, upon informed patient consent (Table 1). ALS diagnosis was performed by licensed neurologists specialized in motor neuron disease, using revised El Escorial criteria. None of the samples exhibited visible blood contamination. Samples were spun at 1750 g at 4 °C for 10 min to remove cells and debris, then aliquotted and stored in low protein-binding polypropylene tubes at − 80 °C within 2 h of collection.

**Table 1:** CSF sample donor demographics

	Control (n = 87)	ALS (n = 236)
Age at donation (mean ± SD)	48.4 ± 16.3	53.7 ± 14.2
Sex (F/M)	45/42	70/166
Disease type (sporadic/familial)	–	205/31

### CSF hemoglobin and total protein measurements

CSF samples were thawed on ice immediately prior to use, and free hemoglobin levels measured using a human hemoglobin ELISA kit, according to manufacturer’s instructions (Cat# E80-136, Bethyl Laboratories). Briefly, 100 µL of capture antibody (1:100 dilution) was added to each well of a 96-well plate and incubated overnight at 4 °C. The plate was then washed and 100 µL of CSF (diluted 1:10 in blocker casein (Thermo Scientific) in Tris-buffered saline) was added to each plate well for 1 h at room temperature. The plate was washed and 100 µL of detection antibody (1:40,000 dilution) added to each well for 1 h at room temperature. After final washes, 3,3',5,5'-tetramethylbenzidine substrate solution was added to each well for 15 min in the dark at room temperature, and the reaction quenched by adding 100 µL of 1 M HCl. The absorbance was measured at 450 nm using a plate reader. All samples were measured in duplicate, and the final free hemoglobin concentration calculated using a standard curve generated using serially diluted pure human hemoglobin calibrator standard. The limit of detection was 6.25 ng/mL. Total protein concentration in 200 µL CSF was measured using the BCA assay (Thermo Scientific; Rockford, IL).

### Patient spinal cord tissue

Post-mortem human spinal cord tissue was obtained from the Neurological Foundation Human Brain Bank at the Centre for Brain Research, Auckland, New Zealand (Table 2). Consent was obtained from donors and their families prior to death and ethical approval for this study was obtained from The University of Auckland Human Participants Ethics Committee. Clinical diagnosis of ALS was performed by consultant neurologists at Auckland City or Middlemore Hospitals, Auckland, New Zealand. Neuropathological diagnosis was performed by consultant neuropathologists at Auckland City Hospital.

**Table 2:** Post-mortem tissue donor demographics

Group	Case code	Age (y)	Sex	Post-mortem delay (h)	Cause of death
Control (n = 5)	H232	89	F	28.5	Multiple stroke, dementia
	H233	83	F	18	Acute renal failure, bowel cancer
	H234	86	M	3	Stroke, dementia
	H235	83	M	21	Hypothermia
	H236	96	F	15	General inanition
	Mean (± SD)	87.4 ± 5.4	F/M: 3/2	17.1 ± 9.3	

Group	Case code	Age (y)	Sex	Post-mortem delay (h)	sALS/fALS (genotype)	Site of onset
ALS (n = 13)	MN7	55	F	56	sALS	Upper limb
	MN10	46	M	24	sALS	Lower limb
	MN11	77	F	18	fALS	Lower limb
	MN12	49	M	34	sALS	Respiratory
	MN13	55	M	10	sALS	Upper limb
	MN14	59	F	19	fALS	Bulbar
	MN15	54	F	18	sALS	FTD
	MN16	69	M	16.5	sALS	Upper limb
	MN18	53	F	12	fALS (*C9ORF72)*	Lower limb
	MN20	85	M	15	sALS	Lower limb
	MN21	59	F	17.5	fALS	Lower limb
	MN22	64	F	9	sALS	Upper limb
	MN23	79	F	27	fALS (*C9ORF72)*	Lower limb
	Mean (± SD)	61.8 ± 12.2	F/M: 8/5	21.2 ± 12.5		

*sALS* sporadic ALS, *fALS* familial ALS, *FTD* frontotemporal dementia

Control spinal cords were obtained from cadavers fixed by injection of anatomical embalming fluid (Dodge™ Anatomical mix) via the carotid artery; ALS cords were immersion fixed in 15% formalin. All cords were blocked into segments and paraffin embedded. Three spinal cord regions were analyzed: cervical (C8), mid-thoracic (T7-T9), and lumbar (L4/L5). To select the appropriate segmental block for each case, transverse diameters of all segments were measured with Vernier calipers (Additional file 1: Fig. S1a) and compared with population estimates of segmental diameter (Additional file 1: Fig. S1b) [37]. Cords sectioned and labelled according to vertebral level (ending at L1) were wider caudally than those labelled according to neuronal level (ending at S5). Segments were then relabelled for neuronal level using a conversion scheme [37], after which an expected and significant reduction in transverse diameter of the spinal cord was seen at both C5 and L4 in ALS (Additional file 1: Fig. S1c and d).

Because control and ALS spinal cords were differently fixed, we tested whether fixation affected immunohistochemical labelling. Alternating spinal cord segments from each of the three spinal cord levels of interest from a single ALS case (age at death, 58 y; post-mortem delay, 19 h; sex, F; *C9ORF72*-negative) were immersion fixed in anatomical embalming fluid (Dodge™) or 15% formalin. Dodge™-fixed tissue edges demonstrated higher levels of anti-hemoglobin antibody labelling, but there was no difference in immunogenicity elsewhere in the tissue with anti-hemoglobin, pTDP-43, or SMI-32 antibodies, or lectin (Additional file 1: Fig. S2).

### Fluorescent immunohistochemistry

Serial spinal cord tissue sections from paraffin-embedded blocks were cut with a microtome in the transverse plane at 10 µm thickness and mounted onto Superfrost Plus slides (Thermo Fisher). Following desiccation for > 1 week at room temperature, three sections per block, each separated by 100 µm (i.e. every 10th section) in order to sample different motor neurons, were processed for immunohistochemistry. Slides were heated to 60 °C for 1 h on a hot plate before dewaxing through an alcohol-xylene-water series: 100% xylene, 2 × 30 min; 100% ethanol, 2 × 15 min; 95%, 85%, 75% ethanol, 2 min each; water 3 × 5 min. Heat-mediated antigen retrieval was performed by immersing slides in sodium citrate buffer pH 6 (Abcam) or Tris-ethylenediaminetetraacetic acid (Tris–EDTA) buffer pH 9 (Abcam) for 2 h in a pressure cooker, followed by 1 × phosphate-buffered saline (PBS) washes, 3 × 5 min, and drawing of wax borders. Sections were permeabilized with PBS-T (0.1% Triton X-100) for 15 min at 4 °C followed by PBS washes 3 × 5 min and blocking in 10% normal donkey serum (NDS) for 1 h. Primary antibodies (AQP4, Sigma-Aldrich AB3594, 1:500; Claudin-5, Abcam Ab131259, 1:1000; Collagen IV, Abcam Ab6586, 1:500; GFAP, DAKO Z0334, 1:2000; Hemoglobin, R&D Systems G-134-C, 1:300; P-glycoprotein, Abcam Ab170904, 1:125; Phospho-TDP-43, Cosmo Bio TIP-PTD-P02, 1:4000; SMI-32, Covance SMI-32R, 1:800; ZO-1, Invitrogen 33–9100, 1:200) and lectin for blood vessels (Biotin-agglutinin lectin *Ulex europaeus*, Sigma L8262, 1:1000) were applied overnight in 1% NDS. No-primary controls received 1% NDS only. Following washing, secondary antibodies (Invitrogen A11058, A21202, or A21206, 1:500) and Cy3- or Cy5-streptavidin for lectin-labelled blood vessels (Invitrogen 434315, 1:500; Jackson Laboratories 016-170-084, 1:500) were applied for 3 h in 1% NDS. Sections were counterstained with Hoechst 33342 nuclear stain for 5 min at 0.5 μg/mL. Where indicated, autofluorescence was quenched with 1 × TrueBlack (Biotium) in 70% EtOH for 30 s at room temperature. Sections were coverslipped with #1.5 coverslips (Menzel-Glaser) using ProLong Diamond Antifade Mountant (Invitrogen). Staining was performed for one section per spinal cord level per case in a single staining run (1 × 3 × 18 = 54 sections per run). This was then repeated twice more, such that run variability did not influence case comparisons, and giving three sections per level per case for image analyses.

### Imaging

Sections co-labelled for hemoglobin, lectin, and SMI-32; or hemoglobin, lectin, and barrier integrity markers; were imaged across the entire section with a MetaSystems VSlide slide scanner at 10×magnification (0.45 NA), using MetaCyte acquisition and stitching software. The Colibri 2 LED light source (Zeiss) was used to acquire DAPI (excitation band 375/38 nm), AlexaFluor 488 (484/25 nm), Cy3 (580/23 nm), and Cy5 (631/22 nm), while the X-Cite light source was used for AlexaFluor 594 (560/40 nm). Phospho-TDP-43 (pTDP-43) pathology in the spinal cord anterior horn was imaged with a Nikon Eclipse N*i*E microscope with a Nikon DS-Ri2 camera using NIS elements (Nikon, Version 4.20) at 20×magnification (0.50 NA). All sections (ALS and control) were imaged with the same settings for each staining combination. Vessel-associated pTDP-43 inclusions were imaged on an Olympus FV1000 confocal microscope at 60× (1.35 NA) magnification.

### Image analysis

The overall image analysis pipeline is shown in Additional file 1: Fig. S3a. SMI-32-immunopositive motor neurons in both entire anterior horns were counted manually using ImageJ (version 1.51p, National Institutes of Health) using the polygon and multipoint tools (two clicks per cell body) based on staining intensity above local background. The number of SMI-32-positive motor neurons was normalized to the area of tissue analyzed (i.e. anterior horn area). An observer blinded to the disease status of the tissues performed a second count using the same methodology but only counting one of the three sections for each level and each case. There was a strong positive linear correlation between motor neuron counts performed by each rater (Additional file 1: Fig. S3b, Pearson r = 0.858, *p* < 0.0001).

Hemoglobin leakage and density of lectin-positive vessels were quantified in an automated fashion using a custom journal for MetaMorph software (version 7.8.10, Molecular Devices). Briefly, each image of the whole spinal cord was separated into spinal cord gray and white matter, excluding the tissue edge, using the draw regions tool. For gray matter, the “Cell Scoring” module was used to measure the integrated intensity of hemoglobin staining which met specified size and intensity criteria (relative to background) and which was also within a defined radius of lectin-positive blood vessels that met separate size and intensity criteria (relative to background). Simultaneously, this module counted the number of vessels that met these criteria (number of vessels analysed per section, 214-3944). For white matter, where vessels are scant, the requirement for the hemoglobin staining to be near to lectin-positive blood vessels was removed, but vessels were still counted (number of vessels analysed per section, 269-5406). For both gray and white matter the integrated intensity of hemoglobin staining and the number of lectin-positive vessels were normalized to the area of tissue analyzed. Automated analyses of hemoglobin leakage were validated by manual grading by an observer blinded to disease status of the tissues. Hemoglobin leakage was graded on composite images of hemoglobin and lectin using a semi-quantitative 3-point grading scale, and showed a positive linear correlation with the automated analysis (Additional file 1: Fig. S3c, Pearson r = 0.640, *p* < 0.0001).

Phospho-TDP-43 (pTDP-43) pathology was quantified automatically using the “Count Nuclei” module within MetaMorph to measure the number of pTDP-43 inclusions in both entire anterior horns that met standardized size and intensity criteria. The number of pTDP-43 inclusions was normalized to the number of motor neurons in the anterior horn area analyzed. An observer blinded to the disease status of the tissues validated the methodology with manual counting of pTDP-43 inclusion number in ImageJ based on staining intensity above local background. This showed a strong positive linear correlation with the automated analysis (Additional file 1: Fig. S3d, Pearson r = 0.964, *p* < 0.0001).

Neurovascular unit components that regulate the BSCB were studied within five of the ALS cases with the highest overall hemoglobin leakage, at the level of cord with greatest leakage for each case (claudin-5, ZO-1, P-glycoprotein, collagen IV; lumbar or thoracic), or within all ALS cases (AQP4, GFAP; thoracic). Vascular or perivascular marker intensity was quantified in an automated fashion using a custom MetaMorph journal. Briefly, adaptive threshold analysis was applied to identify areas of hemoglobin leakage. A ‘vascular mask’ (for claudin-5, ZO-1, P-gp) was created using the adaptive threshold tool to binarize the lectin image. A ‘perivascular mask’ was generated by subtracting the binary lectin mask with holes filled then eroded by 5 µm, from the binary lectin mask dilated 10 µm (for collagen IV) to produce a mask of donut-shaped regions. A ‘glia limitans mask’ was generated by subtracting the binary lectin mask with holes filled, from the binary lectin mask dilated by 20 µm (for AQP4, GFAP). Within each vessel mask, the marker staining intensity within a specified intensity criteria (absolute intensity) was quantified. Each vessel was also scored as either leaked or non-leaked, based on whether the vessel mask was located within the area of the hemoglobin leakage mask. Mean perivascular marker intensity per vessel was then averaged across all vessels for a given region type (number of vessels analyzed per section, claudin-5, 495-2358; ZO-1, 530-2575; P-glycoprotein, 564-2675; collagen IV, 450-2057; AQP4, 368-1311; GFAP, 273-1432).

### Statistical analysis

Statistical analyses were conducted using GraphPad Prism (version 8.0.2). Statistical significance was set at *p* < 0.05. Control and ALS CSF hemoglobin levels, total protein, and hemoglobin normalized to total protein were compared using the Mann–Whitney test. All pooled comparisons between control and ALS spinal cord tissues were performed using Student's *t*-test with Welch's correction. Comparisons between control and ALS cases at each level of the spinal cord (with or without comparisons between segmental levels within control and ALS) were performed using two-way ANOVA with Sidak’s post-test. Comparisons between spinal cord segmental levels within control and ALS were performed using two-way ANOVA with Tukey’s post-test. Comparisons across the spinal cord for ALS alone were performed using one-way ANOVA with Tukey’s post-test. Comparisons between leaked and non-leaked regions in gray and white matter were performed using repeated measures (both factors) two-way ANOVA with Sidak’s post-test. Correlations were analyzed by linear regression and Pearson correlation. Graphs depict individual cases as points (mean of 3 tissue sections per case), overlaid with mean ± standard deviation from the mean (SD) for all cases, unless specified otherwise. Using Grubbs’ test (alpha 0.2), one control case was identified as an outlier for motor neuron number and hemoglobin staining and was thus excluded from all graphical and statistical analyses.

## Results

### Hemoglobin levels in the CSF of living ALS patients

**Fig. 1:**
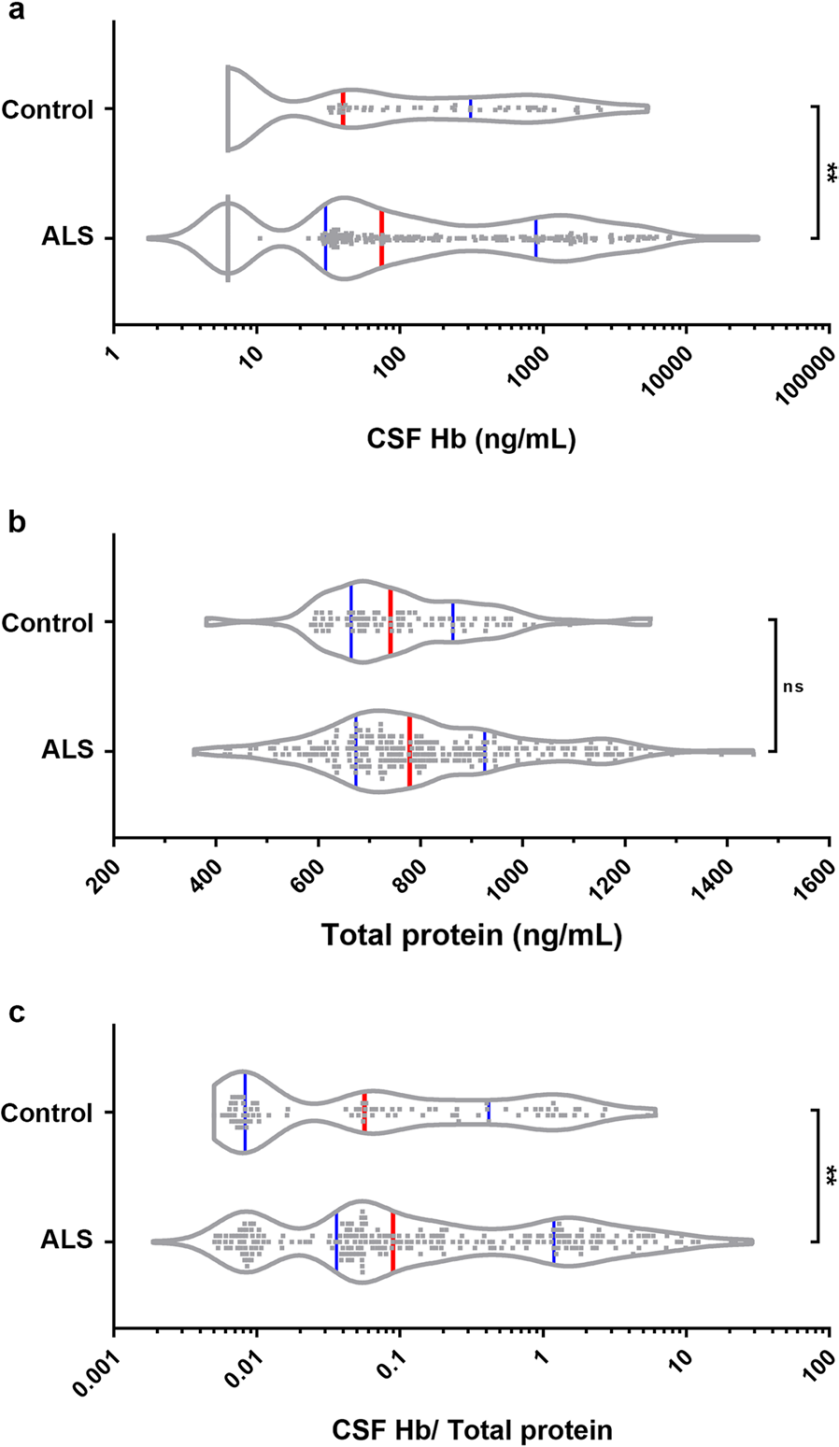
CSF hemoglobin levels in neurologically normal controls and subjects living with ALS. Violin plots (frequency distribution) of CSF hemoglobin concentration (**a**), CSF total protein concentration (**b**), and CSF hemoglobin normalized to total protein (**c**) from patients with ALS (n = 236) compared to control subjects (n = 87). Data shown as median (red bars) with quartiles (blue bars), with statistical significance determined using Mann–Whitney tests. **p ≤ 0.01, ns; not significant

For studies of ALS biomarkers, cerebrospinal fluid (CSF) was banked from individuals living with ALS (sporadic or familial, n = 236) and healthy controls (n = 87) using standard operating procedures established by the Northeast ALS Consortium (NEALS). Hemoglobin levels are routinely measured by ELISA in these samples to assess potential blood contamination of the CSF during the lumbar puncture. However, we noted that samples with very high hemoglobin were predominantly from ALS patients, leading us to examine whether hemoglobin itself may be a disease biomarker. Indeed, CSF hemoglobin levels were significant elevated in patients diagnosed with ALS compared to healthy controls (Median: Control, 39.86 ng/mL; ALS, 74.25 ng/mL). Upper quartile: Control, 310.4 ng/mL; ALS, 889.8 ng/mL, Fig. 1a, *p* = 0.0043). This was not due to increased total protein in the CSF (Median: Control, 741.0 ng/mL; ALS 778.5 ng/mL, Fig. 1b, ns), such that CSF hemoglobin normalized to total protein was still increased in ALS patients compared to controls (Median: Control, 0.056; ALS, 0.089. Upper quartile: Control, 0.416; ALS, 1.184, Fig. 1c, *p *= 0.0067). These data indicate that hemoglobin, which should be restricted to the intravascular compartment, may leak across the blood–brain, blood-spinal cord, or blood-CSF barrier into the CSF compartment of ALS patients.

### Hemoglobin is found in both gray and white matter parenchyma around blood vessels in the ALS spinal cord

**Fig. 2:**
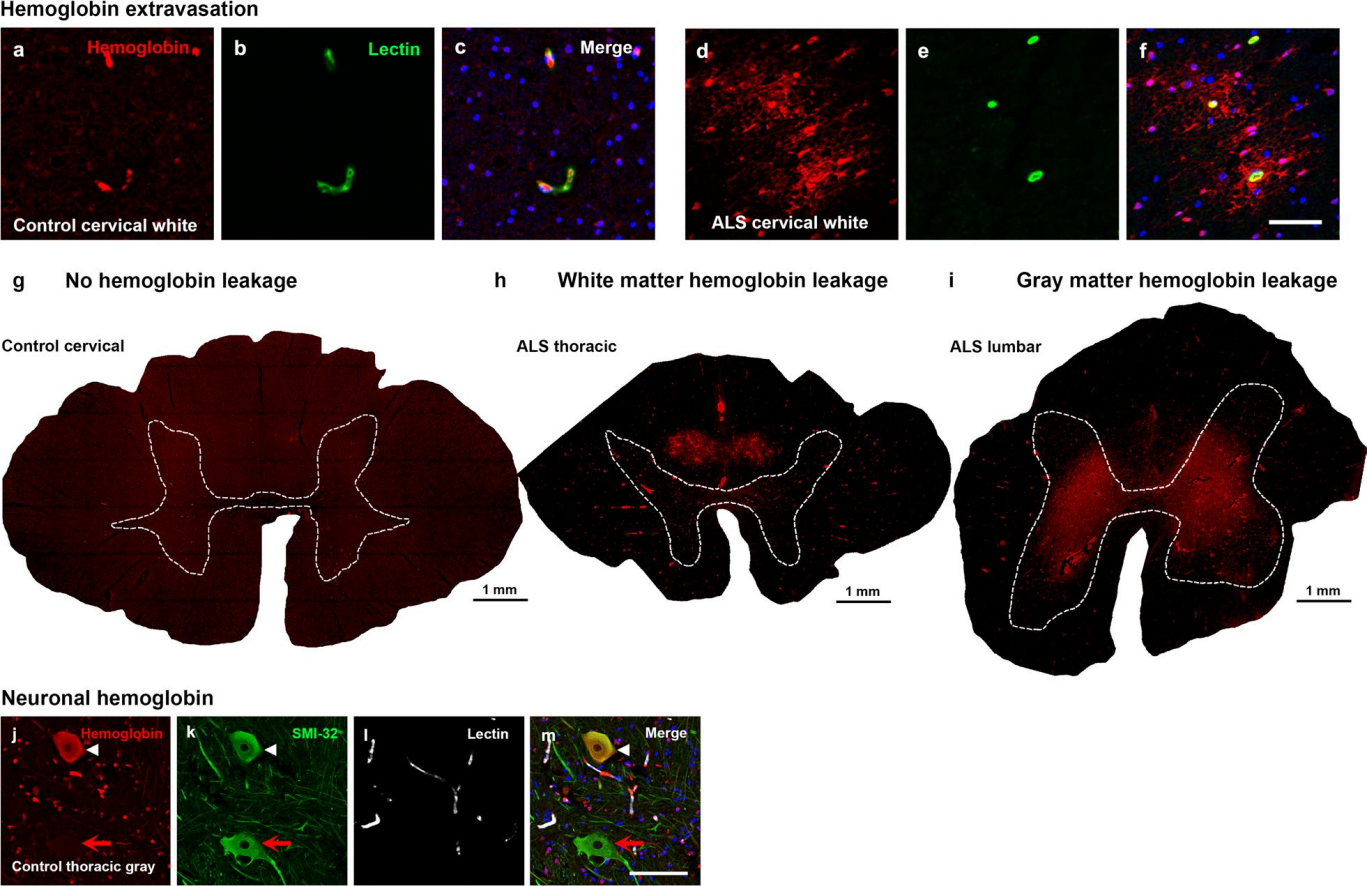
Hemoglobin localization in control and ALS spinal cord. Immunohistochemical staining of hemoglobin extravasation in cervical white matter from control (**a**–**c**) or ALS (**d**–**f**) spinal cord. Hemoglobin immunoreactivity (red) and lectin-positive vessels (green) are shown with a Hoechst nuclear counterstain (blue). Scale bars = 50 µm. Representative images of full spinal cord sections from the cervical level of a control case with no hemoglobin leakage (**g**), the thoracic level of an ALS case with white matter hemoglobin leakage (**h**), and the lumbar level of an ALS case with gray matter hemoglobin leakage (**i**) are shown. Dashed lines show gray matter boundary; scale bar = 1 mm. Occasional hemoglobin staining of SMI-32-positive anterior horn motor neurons was also observed (**j**–**m**). White arrowhead indicates a SMI-32-positive hemoglobin-positive motor neuron, where the red arrow indicates an SMI-32-positive hemoglobin-negative motor neuron. Scale bars = 50 µm

To investigate leakage across the blood-spinal cord barrier specifically, cervical, thoracic, and lumbar control and ALS spinal cord tissue were immunolabelled for hemoglobin. While hemoglobin was localized predominantly within blood vessels in control spinal cords (Fig. 2a–c), there was extravascular hemoglobin staining in most ALS spinal cords (Fig. 2d–f). Extravascular hemoglobin in ALS cases showed a radial distribution around blood vessels, implicating vessel leakage as its source (Fig. 2f). No hemoglobin leakage was evident in control cases, although non-specific background labelling intensity tended to be high (Fig. 2g). In white matter, hemoglobin leakage was seen focally in the dorsal aspects (Fig. 2h) and was present in 12/13 ALS cases (92%), while in gray matter the pattern of leakage was diffuse and mostly dorsal of the transverse midline (Fig. 2i) and was observed in 10/13 ALS cases (77%). In some control and ALS cases, neuronal hemoglobin labelling was observed in neurofilament H (SMI-32)-positive anterior horn motor neurons (Fig. 2j–m), as is seen in human cortical neurons [38, 39], or in cells in the dorsal white matter of the spinal cord which were negative for markers of microglia (Iba1, CD14) or astrocytes (GFAP) (images not shown).

### Hemoglobin leakage varies along the ALS spinal cord axis being most severe at the mid-thoracic level

**Fig. 3:**
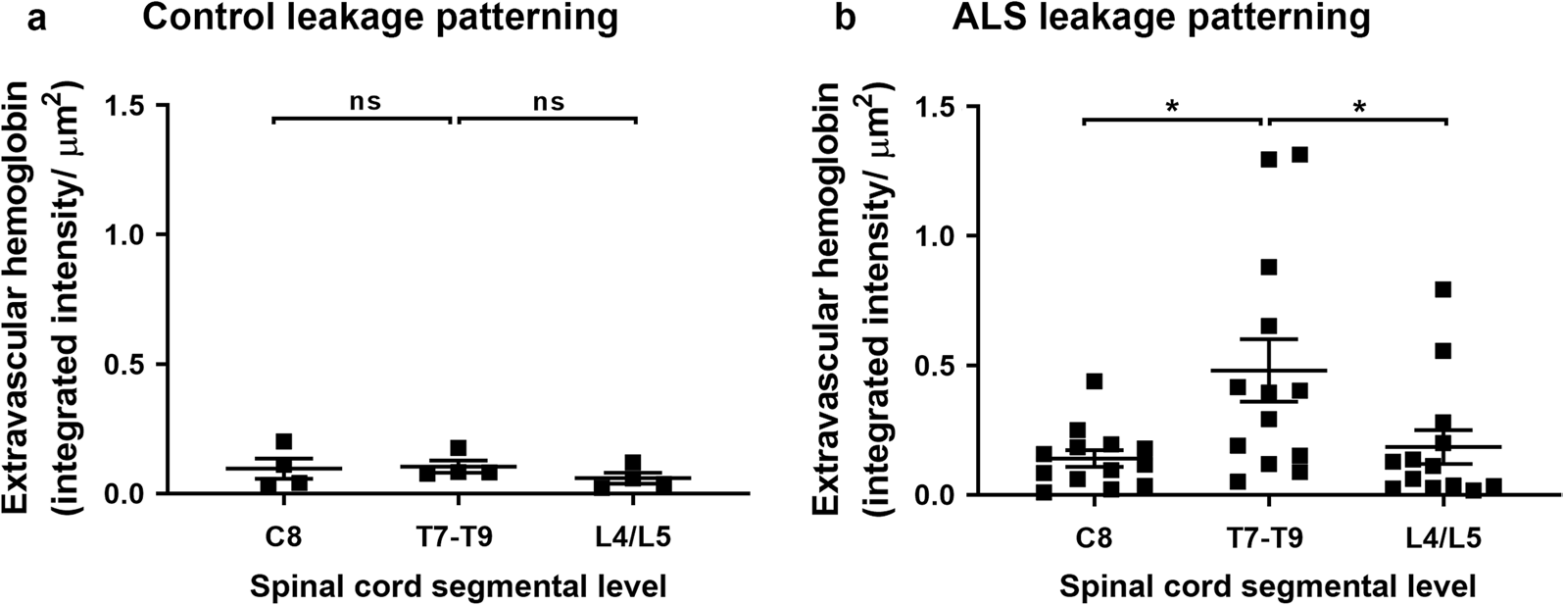
Patterning of hemoglobin leakage along the spinal cord axis. Quantification of extravascular hemoglobin at individual segmental levels C8, T7–T9 and L4/L5 in control (**a**) and ALS (**b**) cases. Data shown as mean ± SD (control n = 4, ALS n = 13), with statistical significance determined using two-way ANOVA with Tukey’s post-test. *p ≤ 0.05; ns, not significant

We next conducted automated image analysis of the pattern of hemoglobin leakage. As the control and ALS tissue was processed using different fixation methods, we first confirmed that fixation did not affect the immunoreactivity of hemoglobin, SMI-32, or pTDP-43 (Additional file 1: Fig. S3; refer to methods). To further ensure our analysis was not confounded by fixation effects, our subsequent analysis focused on the pattern of hemoglobin leakage along the cord within rather than between control and ALS cohorts. Within the control cohort, we observed uniform extravascular hemoglobin at all three levels of the spinal cord (Fig. 3a, ns). However, in ALS cases, patterning was observed along the spinal cord axis with leakage more severe at the mid-thoracic level than the cervical (Fig. 3b,  *p *= 0. 005) or lumbar levels (Fig. 3b, *p *= 0.017). This pattern was maintained regardless of the site of symptom onset (Additional file 1: Fig. S4a).

**Fig. 4:**
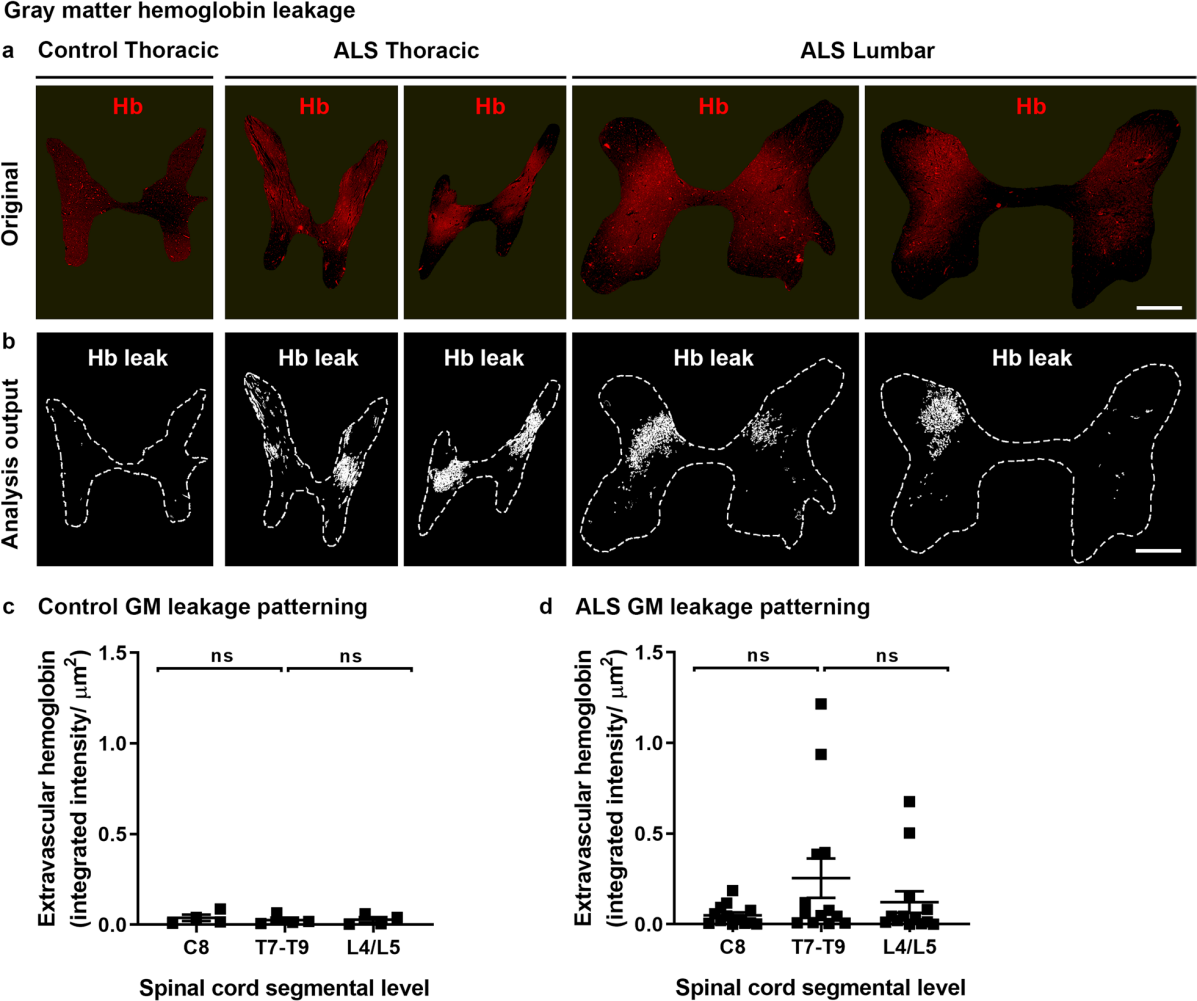
Gray matter hemoglobin leakage along the spinal cord axis. Immunohistochemical labelling (**a**) and automated analysis outputs (**b**) of extravascular hemoglobin in control and ALS spinal cord gray matter. Scale bars = 1 mm. Quantification of extravascular hemoglobin in gray matter at individual segmental levels C8, T7–T9 and L4/L5 in control (**c**) and ALS (**d**) cases. Data shown as mean ± SD (control n = 4, ALS n = 13), with statistical significance determined using two-way ANOVA with Tukey’s post-test. ns, not significant

We next examined hemoglobin leakage in the gray and white matter separately. Gray matter leakage in ALS spinal cords was bilateral and in the mid-dorsal aspect of the gray matter, being mainly absent from the anterior horns, the spinal canal, or laminae I or II (Fig. 4a). Gray matter leakage was detected by automated analysis (as per Fig. 4b). Within the control cohort, gray matter leakage was low in all cases and consistent across all three spinal cord levels (Fig. 4c, ns). In contrast, in the ALS cohort gray matter leakage was more variable between cases, and highest at the mid-thoracic level, although this did not reach significance (Fig. 4d, ns).

**Fig. 5:**
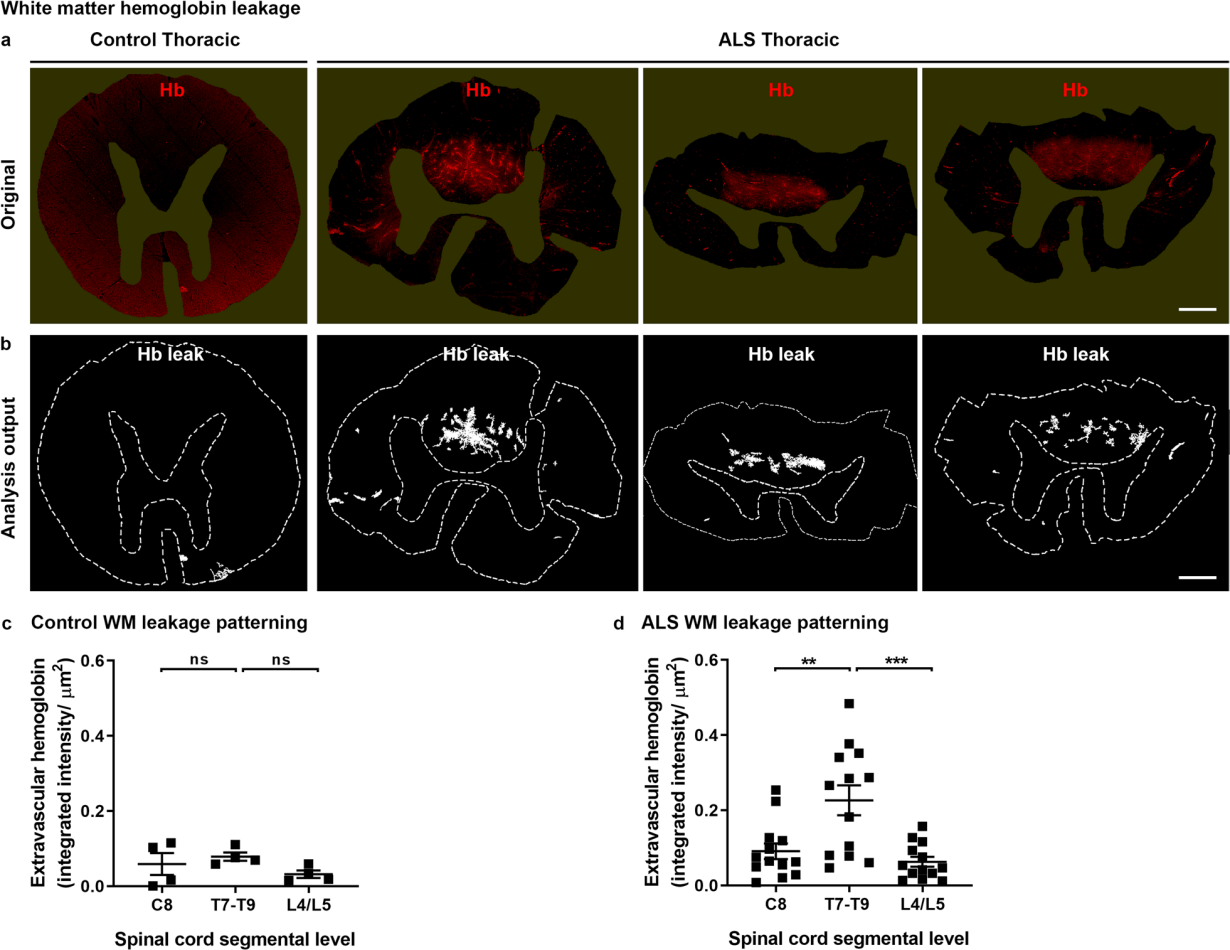
White matter hemoglobin leakage along the spinal cord axis. Immunohistochemical labelling (**a**) and automated analysis outputs (**b**) of extravascular hemoglobin in control and ALS spinal cord white matter. Scale bars = 1 mm. Quantification of extravascular hemoglobin in gray matter at individual segmental levels C8, T7–T9 and L4/L5 in control (**c**) and ALS (**d**) cases. Data shown as mean ± SD (control n = 4, ALS n = 13), with statistical significance determined using two-way ANOVA with Tukey’s post-test. ***p ≤ 0.001; **p ≤ 0.01; ns, not significant

White matter hemoglobin leakage in ALS spinal cords was predominantly dorsomedial (Fig. 5a, b). Within the control cohort, a low level of extravascular hemoglobin was observed at all three spinal cord levels (Fig. 5c, ns), whereas in ALS cases white matter hemoglobin leakage at the mid-thoracic level was significantly greater than at the cervical (Fig. 5d, *p *= 0.0009) or lumbar levels (Fig. 5d, *p* < 0.0001).

### Hemoglobin leakage occurs in regions distinct from motor neuron loss and ALS proteinopathy

Considering this unique pattern of hemoglobin leakage in the ALS spinal cord, we sought to determine whether the increased leakage regionally correlated with motor neuron loss or pathological aggregate load. To do this, we first quantified motor neuron cell density and pTDP-43 load in the anterior horn of ALS versus control spinal cords.

**Fig. 6:**
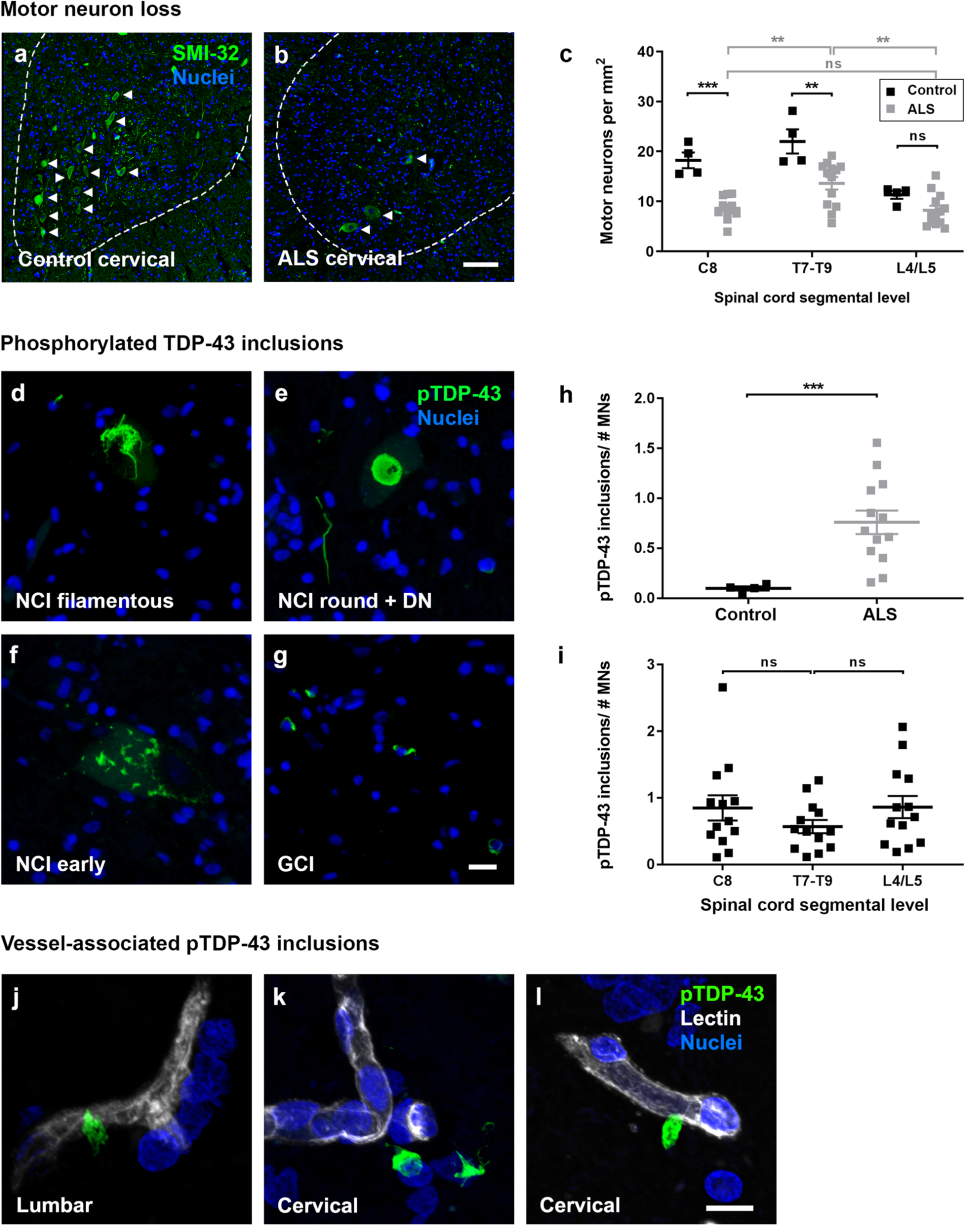
Motor neuron loss and pTDP-43 pathology along the spinal cord axis. Immunolabelling of spinal cord motor neurons with SMI-32 (green) and a Hoechst nuclear counterstain (blue) in control (**a**) and ALS (**b**) cervical spinal cord. Motor neuron numbers per area of anterior horn were manually quantified at individual segmental levels C8, T7-T9, and L4/L5 in control and ALS spinal cords (**c**). Immunohistochemical labelling of phosphorylated TDP-43 in ALS spinal cords; filamentous (**d**), round (**e**), or early (**f**) neuronal cytoplasmic inclusions (NCI) or dystrophic neurites (DN), and glial cytoplasmic inclusions (GCI) (**g**). Scale bars = 10 µm. Automated counting of pTDP-43 inclusions of 8 µm diameter or greater detected neuronal inclusions but rarely glial inclusions and normalized to the number of motor neurons averaged across cervical C8, thoracic T7-T9, and lumbar L4/L5 for control and ALS spinal cords (**h**). Phospho-TDP-43 inclusions/number of motor neurons were then investigated at individual segmental levels C8, T7-T9, and L4/L5 for ALS spinal cords (**i**). All data shown as mean ± SD, control n = 4, ALS n = 13, with statistical significance determined using two-way ANOVA with Tukey’s post-test (**c**, **i**). or student’s t test with Welch’s correction (**h**). ****p ≤ 0.0001; ***p ≤ 0.001; ns, not significant. Rare pTDP-43 inclusions (green) adjacent to or associated with lectin-positive blood vessels (white) in ALS lumbar and cervical spinal cord (**j**–**l**). Scale bars = 10 µm

Immunolabelling for the neurofilament heavy chain protein using the SMI-32 antibody identified neuronal somata and processes throughout the gray matter, including motor neurons of the anterior horns (Fig. 6a and b). Manual counting of SMI-32-positive motor neurons specifically in the anterior horn revealed motor neuron loss in ALS across all three levels of the cord examined (Fig. 6c); with statistically significant loss compared to controls at C8 (*p* = 0.0002) and T7-T9 (*p* = 0.0014), and significantly more neurons remaining in ALS thoracic than either ALS cervical (*p* = 0.0075) or ALS lumbar cord (*p* = 0.0029). Site of symptom onset (upper limb; lower limb; other (respiratory, bulbar, frontotemporal dementia)) did not influence the pattern of motor neuron loss along the spinal cord axis (Additional file 1: Fig. S4b).

We next examined the presence and load of the hallmark pTDP-43 deposits in the spinal cord. pTDP-43 was detected in all 13 ALS spinal cords, in the form of filamentous, round, or neuronal cytoplasmic inclusions (NCI), dystrophic neurites (DN) and glial cytoplasmic inclusions (GCI) (Fig. 6d–g). Our automated detection of pTDP-43 was set to count inclusions of 8 µm diameter or greater, which identified the majority of neuronal inclusions but few small glial inclusions. Automated analysis showed that pTDP-43 inclusions were selective for ALS (Fig. 6h, *p* = 0.0001), and that pTDP-43 inclusion load in ALS cases was frequently higher in the cervical and lumbar regions, and lower in the mid-thoracic region (Fig. 6i, ns), regardless of the site of symptom onset (Additional file 1: Fig. S4c). Phospho-TDP-43 inclusion load did not predict motor neuron loss in individual cases when the average values over all three levels of the cord were examined (Additional file 1: Fig. S4d). However, phospho-TDP-43 inclusion load did predict motor neuron loss when the cervical cord was examined of upper-limb onset cases (Additional file 1: Fig. S4e, n = 4, Pearson r = − 0.976, *p* = 0.024).

It is important to note that two ALS cases included in this study were known to be positive for *C9ORF72*-repeat expansion mutations. Therefore, in addition to TDP-43 inclusions, we also investigated the presence of dipeptide repeat inclusions in the cervical and thoracic spinal cord. In both cases, polyGA inclusions were extremely rare, and across four sections studied, only one polyGA inclusion was found, which was also positive for the ubiquitin-binding protein, p62 (data not shown). Given the scarcity of spinal cord dipeptide repeat inclusions, as reported by others [40], we did not map these in finer detail.

Overall, in the ALS spinal cord, significant motor neuron loss occurs in the cervical and thoracic segments, and the highest pTDP-43 aggregation occurs in the cervical and lumbar segments. In contrast, hemoglobin leakage is highest within the thoracic segment of the ALS spinal cord. Consistent with hemoglobin leakage occurring in regions distinct from the major motor neuron pathology, hemoglobin load in individual ALS cases did not predict motor neuron number (Additional file 1: Fig. S5a) or pTDP-43 inclusion load (Additional file 1: Fig. S5b). Nor did the extent of hemoglobin leakage correlate with disease duration (Additional file 1: Fig. S5c) or post-mortem delay (PMD) (Additional file 1: Fig. S5d).

Despite hemoglobin leakage occurring in regions distinct from pTDP-43 deposition, we subsequently investigated whether pTDP-43 inclusions were found within or associated with the vasculature. While typical parenchymal pTDP-43 pathology was in the form of neuronal or glial cytoplasmic inclusions that were not associated with the vasculature, extremely rare pTDP-43 inclusions were found adjacent to the vessels (Fig. 6j–l). These vessel-associated pTDP-43 inclusions were found in three cases with high overall pTDP-43 load; in the cervical cord of two cases, and lumbar cord of one case. Given the scarcity of vessel pathology, as described by others [41, 42], we did not further investigate correlations with blood spinal cord leakage.

### No loss of selected endothelial and basement membrane markers but possible differences in astrocyte endfeet in areas of hemoglobin leakage

**Fig. 7:**
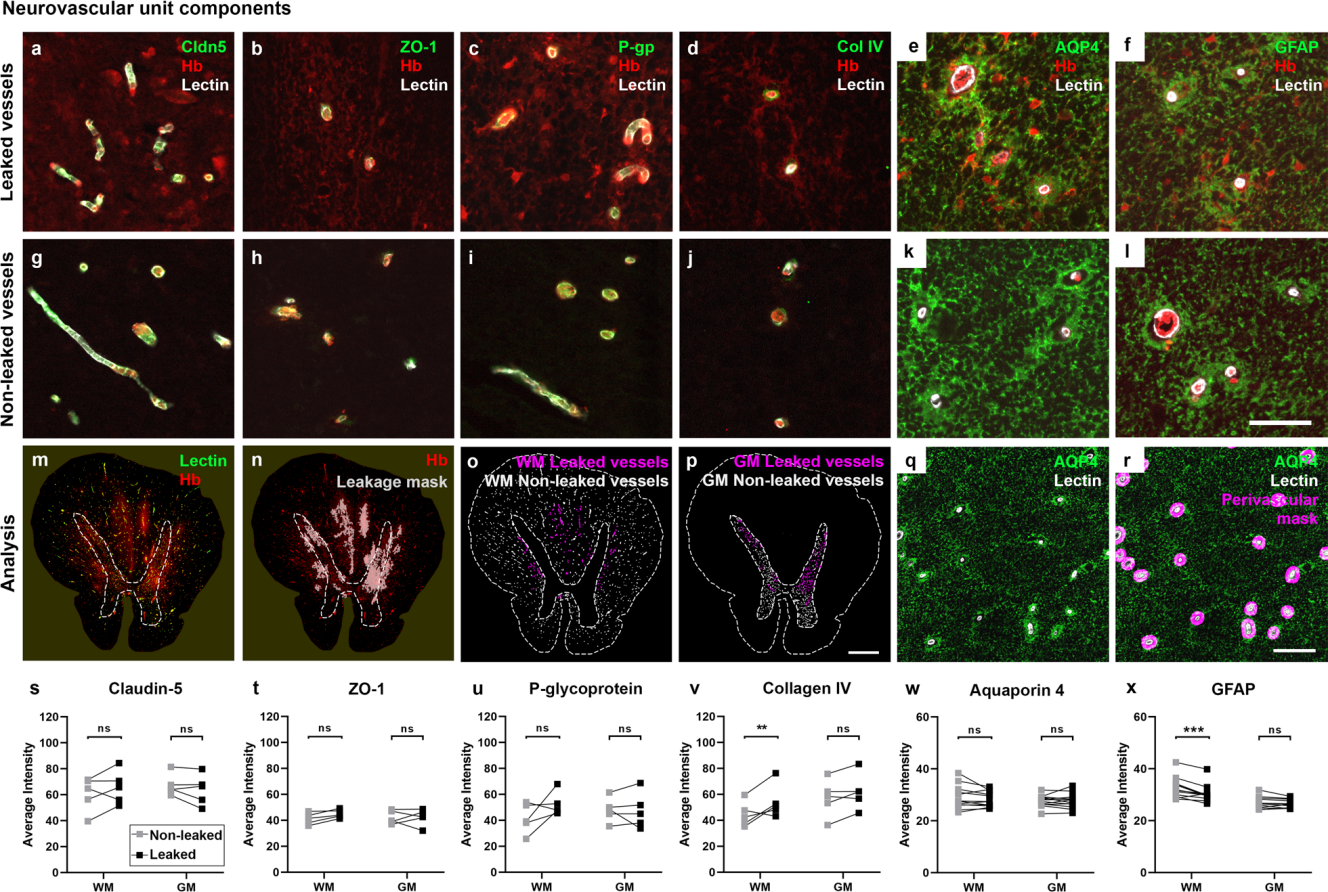
Neurovascular unit component expression in leaked versus non-leaked vessels along the spinal cord axis. Immunohistochemical labelling of neurovascular unit markers in ALS spinal cord (**a**–**l**); tight junctions claudin-5 (**a**, **g**) and ZO-1 (**b**, **h**), efflux pump P-glycoprotein (**c**, **i**), basement membrane marker collagen IV (**d**, **j**), and astrocyte markers aquaporin 4 (**e**, **k**) and GFAP (**f**, **l**), in spinal cord vessels with or without hemoglobin leakage. Scale bar = 50 µm. Automated quantification of average intensity staining of neurovascular unit markers was carried out in all ALS cases (n = 13) or in a subset of ALS cases with high hemoglobin leakage (n = 5) in leaked and non-leaked areas of the white and gray matter of the spinal cord (**m**–**r**). Composite of original images showing anti-hemoglobin immunoreactivity (red) and lectin-positive vessels (green) (**m**) and overlays of hemoglobin leakage analysis output (white, partly transparent) over anti-hemoglobin (red) (**n**). Segmentation of vessels inside (magenta) or outside (white) areas of hemoglobin leakage in the white matter (**o**) or gray matter (**p**). Dashed lines show boundaries. Scale bar = 1 mm. Perivascular astrocyte endfeet staining (green) around lectin-positive vessels (white) in (**q**) was isolated using an automated mask of the glia limitans (**r**). Scale bar = 50 µm. The average intensities of marker staining were measured in leaked and non-leaked vessels of the gray and white matter (**s**–**x**). Data shown as mean ± SD (n = 5 or 13) with statistical significance determined with a two-way repeated-measures ANOVA with Sidak’s post-test. ns = not significant

**Fig. 8:**
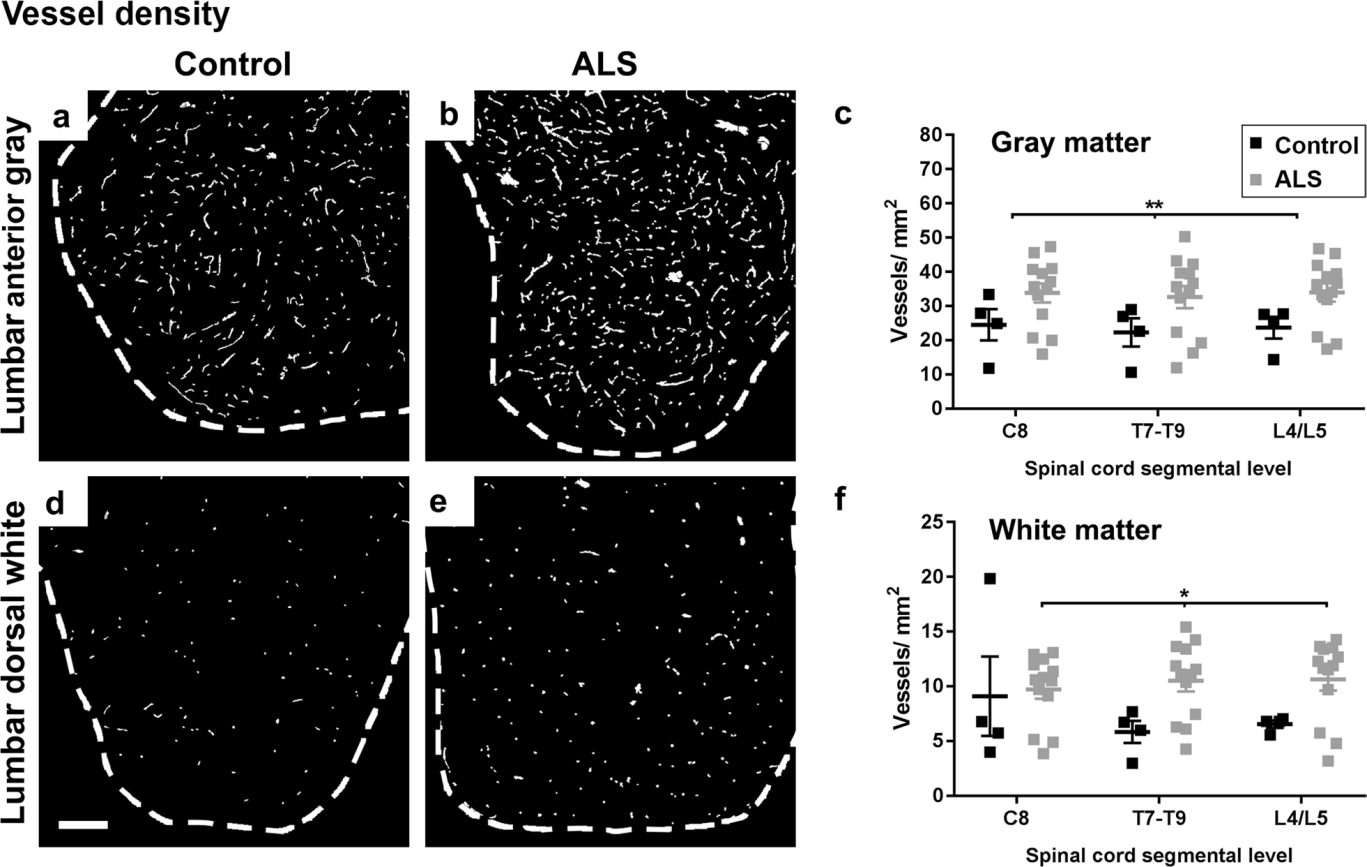
Vessel density along the spinal cord axis. Representative images of lectin-positive blood vessel staining in lumbar anterior gray matter (**a**, **b**) and lumbar dorsal white matter (**d**, **e**) in control and ALS spinal cords. Scale bars = 250 µm. Vessel density was quantified at individual segmental levels C8, T7–T9, and L4/L5 in gray (**c**) and white matter (**f**) of control and ALS spinal cords. Data shown as mean ± SD (control n = 4, ALS n = 13), with statistical significance determined using two-way ANOVA with Sidak’s post-test. **p ≤ 0.01; *p ≤ 0.05

To investigate the mechanisms for pathological passage of hemoglobin across the BSCB in ALS, we quantified changes in neurovascular unit (NVU) components between leaked versus non-leaked vessels. Selected component proteins were stained in a subset of ALS cases with high hemoglobin leakage, at the level of cord with greatest leakage for that case (endothelial tight junction proteins claudin-5 and zonula occludens 1 (ZO-1), efflux transporter P-glycoprotein (P-gp), basement membrane protein collagen IV; lumbar or thoracic), or within all ALS cases (astrocyte filament markers aquaporin 4 (AQP4) and glial fibrillary acidic protein (GFAP); thoracic). All markers were immunodetected within both leaked and non-leaked patches in the spinal cord, as determined by hemoglobin co-labelling (Fig. 7a–l). We used automated detection of patches of hemoglobin leakage, together with manually drawn gray and white matter ‘regions’ and automatically generated vascular, perivascular, or glia limitans masks, to compare the staining intensity of NVU components in leaked and non-leaked regions of the spinal cord, in both the gray and the white matter vessels (Fig. 7m–r). No changes in staining intensity were observed between leaked and non-leaked areas of the spinal cord, in either gray or white matter, for claudin-5, ZO-1, P-gp, or aquaporin 4 (Fig. 7s–u, w, ns). However, in white matter there was a small but significant increase in collagen IV (Fig. 7v, average intensity change 18.1%, *p* = 0.0095) and decrease in GFAP (Fig. 7x, average intensity change 7.4%, *p* = 0.0004) in leaked compared to non-leaked areas. In addition, during our investigations of vessel integrity and hemoglobin leakage, we noted an increase in vessel density in ALS in both gray matter (Fig. 8a–c, *p* = 0.0041) and white matter (Fig. 8d–f, *p* = 0.0126), but with no differences between segmental levels (Sidak’s post tests, ns).

## Discussion

Finding high levels of hemoglobin in the CSF of subjects living with ALS, we examined the relationship between BSCB integrity, endothelial barrier protein expression, motor neuron degeneration and TDP-43 proteinopathy in post-mortem human ALS spinal cord. A key finding of our study was that BSCB compromise marked by hemoglobin deposition in the spinal cord parenchyma around the blood vessels was present along the ALS spinal cord axis. We predicted that BSCB damage would be most severe in the anterior part of the cervical and lumbar cord, given that lower motor neurons in these regions innervate the limbs, which are most commonly affected in ALS [1, 5], and lower motor neuron loss with phosphorylated TDP-43 proteinopathy is greatest in the cervical and lumbar cord [our findings and 5]. Indeed, previous studies found hemoglobin, fibrin, thrombin and IgG extravasation in the cervical spinal cord [17], and fibrin and IgG extravasation in the lumbar spinal cord [18]. The thoracic cord, where the BSCB is ‘tightest’ [16], had not previously been examined. However, we demonstrate that hemoglobin extravasation is also found at the mid-thoracic level, and that this thoracic BSCB leakage is more severe than that in the cervical or lumbar segments. The thoracic spinal cord motor neurons innervate the intercostals and some accessory respiratory muscles, and their degeneration is presumed to be a late-stage feature of disease [43]. Intriguingly, we also find that the most striking hemoglobin extravasation does not occur anteriorly, adjacent to the spinal motor neurons, but in the dorsal aspects of the gray and particularly the white matter. Our findings therefore call into question the paradigm that motor neuron neurodegeneration induces local barrier leakage.

Lower spinal motor neuron somata are predominately located in lamina IX of the spinal cord in the anterior gray matter, with their axons projecting to target muscle fibers via the anterior roots. Yet leaked hemoglobin was detected across both gray and white matter; most severely in the dorsomedial white matter and rarely in the anterior gray matter. Similarly, microhemorrhages in the cervical and lumbar spinal cord of *SOD1* mice were found to be quite uniformly distributed across the gray and white matter, without preference for the anterior gray matter [44]. Therefore, although it is reasonable to predict that motor neuron damage induces neuroinflammation and BSCB permeability to infiltrating immune cells, our data argue against BSCB changes in ALS requiring concurrent nearby neuronal damage.

While motor neuron pathology does not predict areas of BSCB leakage, BSCB leakage may still influence motor neuron degeneration despite their anatomical separation. We have identified CSF hemoglobin as a marker of BSCB leakage that is diffuse and thus has the potential to influence motor neurons at sites distant from its origin. Oxidized extracellular hemoglobin is highly reactive, contributing to oxidative stress, inflammation and tissue damage and yielding toxic metabolites including heme and iron [45]. Spinal cord neurons exhibit toxicity in a *SOD1* mouse model of ALS [27], or when exposed to hemoglobin [46], that is iron-dependent. Hemoglobin can also cause oxidative damage to myelin [47], and remyelination of motor axons in ALS is known to be defective [48, 49]. Therefore, leaked hemoglobin in the CSF and tissue may contribute to damage of axonal pathways locally, and motor neuron somata distantly, although our findings best align with a model where cell autonomous dysfunction linked to TDP-43 proteinopathy is the key initiator of motor neuron degeneration.

TDP-43 aggregate burden has been demonstrated in several large studies to predict neurodegeneration in ALS in both brain and spinal cord [4, 5, 33]. In spinal cord, Brettschneider et al. found pTDP-43 load to be remarkably consistent at all levels as we report here, with the exception of a very modest increase at cervical levels in upper limb-onset cases only [5]. In contrast, motor neuron loss in that study was substantially greater in the cervical regions for upper limb-onset cases, and the lumbar region for lower limb-onset cases [5]. In our study of a mixed-onset ALS cohort, motor neuron loss was not significant in lumbar cord compared to controls, which may relate to the older ages of our controls [50, 51]. However, in agreement with Brettschneider et al., our study found significantly fewer lower motor neurons remaining in the cervical and lumbar, than thoracic anterior horns. In addition, our analysis of upper limb-onset cases at the cervical spinal cord level supports the hypothesis that a greater burden of pTDP-43 in remaining motor neurons predicts greater motor neuron loss. Even small increases in TDP-43 expression in model systems are sufficient to recapitulate ALS phenotypes [52], so although pTDP-43 aggregates in ALS may not themselves be toxic, they indicate the accumulation of a range of TDP-43 protein species [53] which can drive motor neuron degeneration.

Our study has determined the patterning of BSCB leakage and neurovascular unit component expression across gray and white matter and along the spinal cord axis of ALS cases. It should be noted that we did not directly compare the amount of leakage between control and ALS cases as the spinal cords were preserved using different fixatives. We cannot rule out the possibility that different fixatives could confound comparisons between cases, but we demonstrate that all antibodies we used showed comparable labelling of tissue fixed with Dodge™ embalming fluid or 15% formalin from the same ALS case. Indeed, increased hemoglobin leakage in ALS compared to controls has been extensively reported previously for humans and transgenic ALS animal models [11, 17, 49, 54], and is supported by our finding of elevated CSF hemoglobin in living ALS patients.

Using our within-case analysis approach we found no change between leaked and non-leaked regions of individual ALS cords, in endothelial junction proteins claudin-5 and ZO-1 or the efflux protein P-glycoprotein, or in the perivascular astrocyte endfeet marker aquaporin 4, that regulate BSCB tightness. Control cords were not examined. Previous studies found changes in these neurovascular unit components between transgenic ALS models or human patients and their respective controls, and it is presumed that component changes underpin increased permeability. For instance, in post-mortem ALS patient spinal cord, endothelial cells showed reduced expression of claudin-5 [18], ZO-1 [18, 31], and occludin [31], and increased expression of P-glycoprotein [55], compared to controls. However, there are some disparities in the literature; tight junctions were reported to be morphologically normal in the spinal cord in humans with ALS [29] and a TDP-43 conditional knockout mouse [56].

It is important to note that previous studies have not specifically compared leaked and non-leaked vessels as we report here. Our vessel-by-vessel within-case analyses sought not to re-examine whether neurovascular unit components are changed in ALS overall, but to determine the relationship between vessel leakage and component expression. Where we did not see changes in components reported previously to change, such changes may be temporally distinct from the hemoglobin leakage we detect at the end of life.

Notably, we found a modest (18.1%) increase in basement membrane collagen IV surrounding leaked vessels in white matter. Previously, collagen IV in ALS has been reported to either accumulate [18, 29] or decrease [30, 57]. We also detected a small (7.4%) decrease in the astrocyte intermediate filament GFAP in the perivascular region of leaked compared to non-leaked vessels in the white matter. Detachment of GFAP-positive astrocyte end feet from the vasculature has been suggested to occur in human ALS [30] and in a SOD1 transgenic model [58]. However, because AQP4 was not different in leaked vessels, our data do not support end feet detachment. Instead, white matter vessels that leak in ALS may be differentially invested by GFAP-expressing fibrous astrocyte processes [59]. This could be due to the reduced tendency of spinal cord dorsal white matter towards GFAP-positive astrogliosis in ALS [60], or enhanced vulnerability to leakage of vessels encircled by astrocytes with lower GFAP expression [61].

We identified an increase in vessel density in ALS in the gray matter, at all three levels of the cord tested. Increased vascular density in ALS gray matter has been reported previously at the lumbar level [18, 62], particularly in the anterior gray matter [62], but was not seen at the cervical level [18]. Microvessel density changes are likely driven by hypoxia in ALS, given they are mitigated by artificial respiratory support [62], so the finding of increased vessel density in the cervical gray matter in our study but not [18] may be due to differential use of ventilation in the examined patient cohorts. In addition, our use of a vessel-specific lectin afforded us detection of neovascularization independent of basement membrane deposition (collagen) [18] or cell adhesion properties (CD34) [62] of those new vessels. We are the first to report an increase in vessel density in ALS white matter at cervical, thoracic, and lumbar levels. Our findings and those previously suggest that aberrant angiogenesis is a consistent feature in ALS spinal cord and may account for leakage.

We found significantly higher levels of hemoglobin in CSF from people living with ALS. This finding alone cannot distinguish between alterations in CSF production, or disruption at the blood-CSF, blood–brain or blood-spinal cord barriers. However, others also report elevated hemoglobin subunits alpha and beta in ALS CSF compared to controls, in the context of both increases and decreases in other CSF proteins [21]. Taken together with our finding of perivascular hemoglobin deposition in the spinal cord parenchyma, it is reasonable to postulate that elevated CSF hemoglobin in ALS derives at least in part from increased permeability of the BSCB, such that interstitial fluid drainage would enrich the CSF with hemoglobin.

We propose that BSCB breakdown occurs independent of motor neuron pathology in ALS. Several rodent models of ALS show BSCB breakdown prior to motor neuron loss [30, 49], or occurring transiently then resolving [56]. Barrier leakage in human Alzheimer’s disease is also independent of either neurodegeneration or the hallmark proteinopathy, although increased BBB permeability predicts early cognitive dysfunction [48]. Whether leaked hemoglobin in the CSF and tissue can induce symptoms in ALS, and if so at what stage in disease, warrants further study. However, the inverse regional relationship seen here between motor neuron pathology and BSCB leakage in ALS supports the view that cell-autonomous dysfunction linked to TDP-43 proteinopathy, and not BSCB dysfunction, is the major driver of motor neuron degeneration.

## Conclusions

Together our findings suggest that BSCB breakdown varies along and across the spinal cord axis, in a pattern that is independent of that of lower motor neuron death or TDP-43 proteinopathy. BSCB leakage in human spinal cord is therefore unlikely to be caused by lower motor neuron degeneration, and similarly lower motor neuron degeneration is likely driven predominantly by factors other than BSCB disruption.

## Supplementary Information

**Additional file 1: Figure S1.** Spinal cord transverse diameters. **Figure S2.** Immunogenicity of spinal cord was equivalent between Dodge^TM^ and formalin fixation. **Figure S3.** Imaging and quantification workflow. **Figure S4.** Patterning of neither extravascular hemoglobin nor motor neuron pathology were affected by site of ALS onset; pTDP-43 inclusion load predicted motor neuron loss only in cervical cord of upper-limb onset cases. **Figure S5.** Extravascular hemoglobin did not correlate with motor neuron pathology, disease, or spinal cord tissue collection delay. **Table S1.** Review of blood-brain and blood-spinal cord barrier disruption in human ALS studies.

## Data Availability

The datasets used and/or analyzed during the current study available from the corresponding author on reasonable request.

## Abbreviations

ALS: Amyotrophic lateral sclerosis
AQP4: Aquaporin 4
BBB: Blood–brain barrier
BSCB: Blood-spinal cord barrier
CSF: Cerebrospinal fluid
DN: Dystrophic neurites
GCI: Glial cytoplasmic inclusions
GFAP: Glial fibrillary acidic protein
fALS: Familial ALS
FTD: Frontotemporal dementia
IgG: Immunoglobulin G
NA: Numerical aperture
NCI: Neuronal cytoplasmic inclusions
PBS: Phosphate-buffered saline
P-gp: P-glycoprotein
pTDP-43: Phosphorylated TDP-43
sALS: Sporadic ALS
SD: Standard deviation from the mean
SMI-32: Monoclonal anti-neurofilament H
TDP-43: Transactive response DNA-binding protein of 43 kDa
ZO-1: Zonula occludens-1
