# Supplementary material for: Blood-spinal cord barrier leakage is independent of motor neuron pathology in ALS
Source: Acta Neuropathol Commun. 2021 Aug 26;9:144. doi: 10.1186/s40478-021-01244-0 (PMC8393479; doi:10.1186/s40478-021-01244-0)
Supplement: Supplementary file 1 — Additional file 1: Figure S1. Spinal cord transverse diameters. Figure S2. Immunogenicity of spinal cord was equivalent between DodgeTM and formalin fixation. Figure S3. Imaging and quantification workflow. Figure S4. Patterning of neither extravascular hemoglobin nor motor neuron pathology were affected by site of ALS onset; pTDP-43 inclusion load predicted motor neuron loss only in cervical cord of upper-limb onset cases. Figure S5. Extravascular hemoglobin did not correlate with motor neuron pathology, disease, or spinal cord tissue collection delay. Table S1. Review of blood-brain and blood-spinal cord barrier disruption in human ALS studies. [file 40478_2021_1244_MOESM1_ESM.docx]

**Blood-spinal cord barrier leakage is independent of motor neuron pathology in ALS**

**Supplementary Figures and Table**

Sarah Waters‡^1,3^, Molly E. V. Swanson‡^2,3^, Birger V. Dieriks^2,3^, Yibin Zhang^1,3^, Natasha L. Grimsey^1,3^, Helen C. Murray^2,3^, Clinton Turner^3,4^, Henry J. Waldvogel^2,3^, Richard L.M. Faull^2,3^, Jiyan An^5^, Robert Bowser^5,^ Maurice A. Curtis^2,3^, Mike Dragunow^1,3^, Emma Scotter^1,3^

1. Department of Pharmacology and Clinical Pharmacology, University of Auckland, Auckland, New Zealand
2. Department of Anatomy and Medical Imaging, University of Auckland, Auckland, New Zealand
3. Centre for Brain Research, University of Auckland, Auckland, New Zealand
4. LabPlus, Auckland City Hospital, Auckland, New Zealand.
5. Departments of Neurology and Neurobiology, Barrow Neurological Institute, 85013 Phoenix, Arizona, USA.

‡ These authors contributed equally to this work.

*Corresponding authors

**
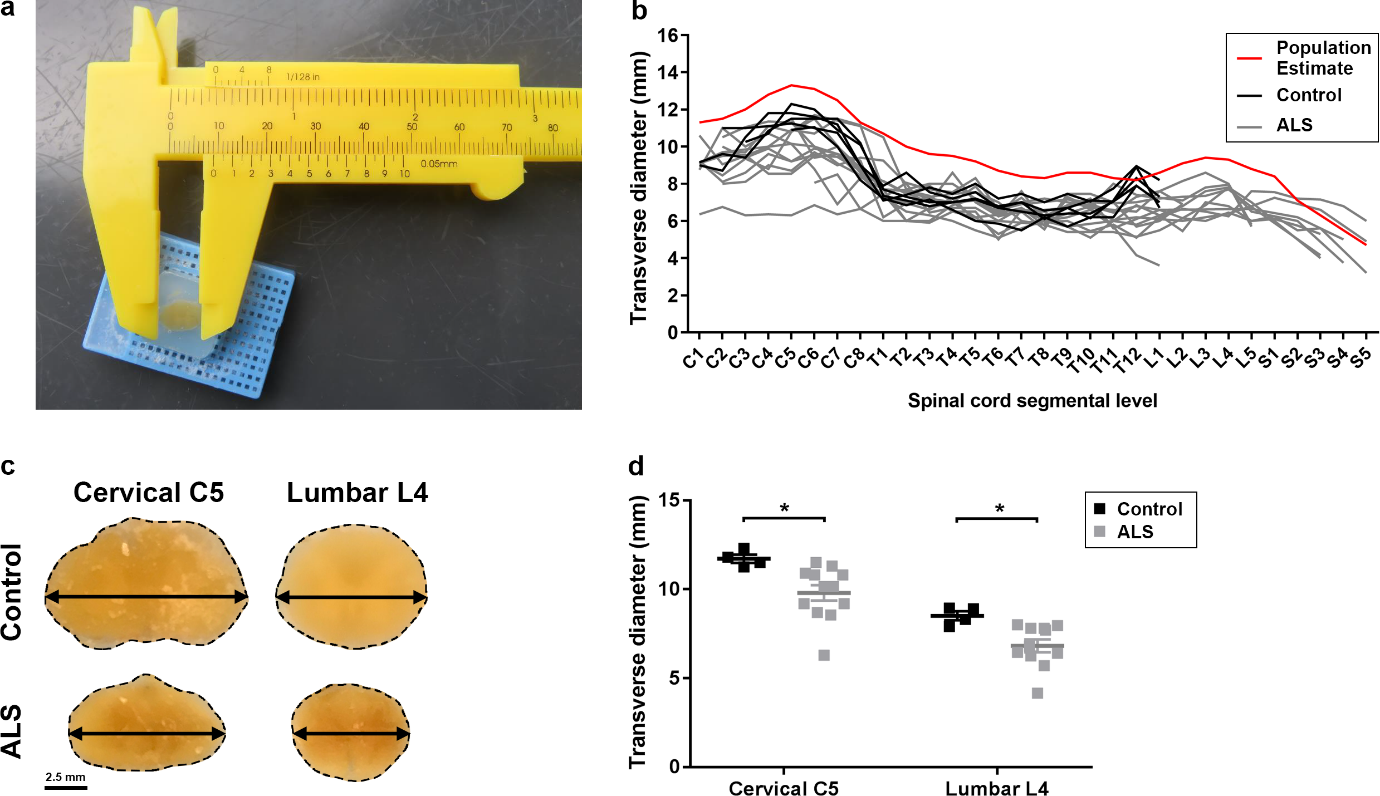
**

**Supplementary Figure 1. Spinal cord transverse diameters**

(**a**) Vernier callipers used to measure spinal cord segment transverse diameters (to nearest 0.05 mm). (**b**) Transverse diameters of control and ALS spinal cords prior to conversion, demonstrating the need for vertebral-to-neuronal segment labelling conversion. Population estimate (red) from [1]. Dashed line indicates the control case that was an outlier for motor neuron counts and hemoglobin staining. (**c**) Photomicrographs of representative paraffin-embedded control and ALS spinal cord segments. Scale bars = 2.5 mm. (**d**) Transverse diameters of cervical segment C5 and lumbar segment L4 for control and ALS cases.

**
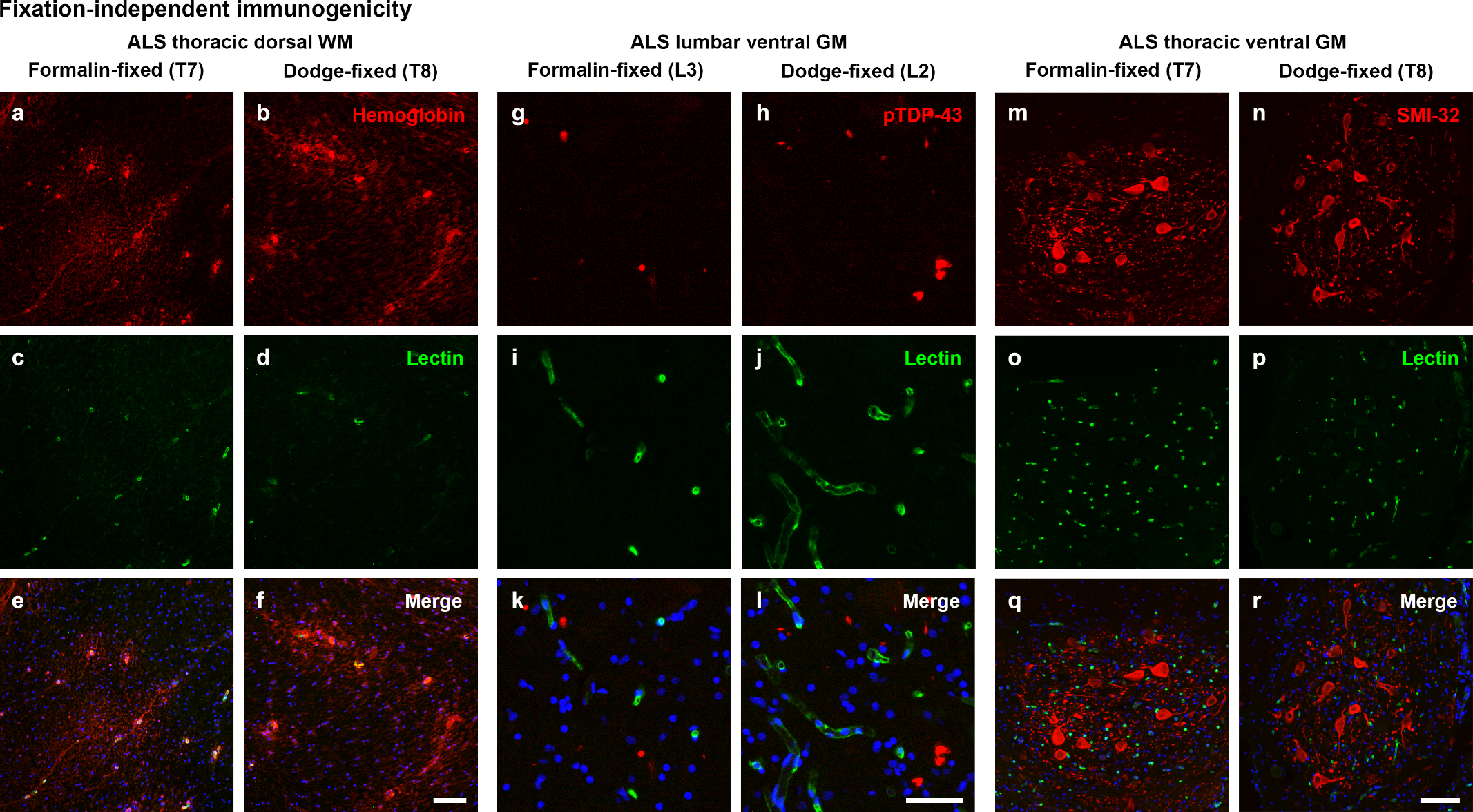
**

**Supplementary Figure 2. Immunogenicity of spinal cord was equivalent between Dodge^TM^ and formalin fixation**

Immunolabelling of hemoglobin and lectin (**a-f**), pTDP-43 and lectin (**g-h**), and SMI-32 and lectin (**m-r**) in adjacent formalin- and dodge-fixed spinal cord blocks from a single ALS case. Scale bars = 100 µm (**a and c**), 50 µm (**b**).

**
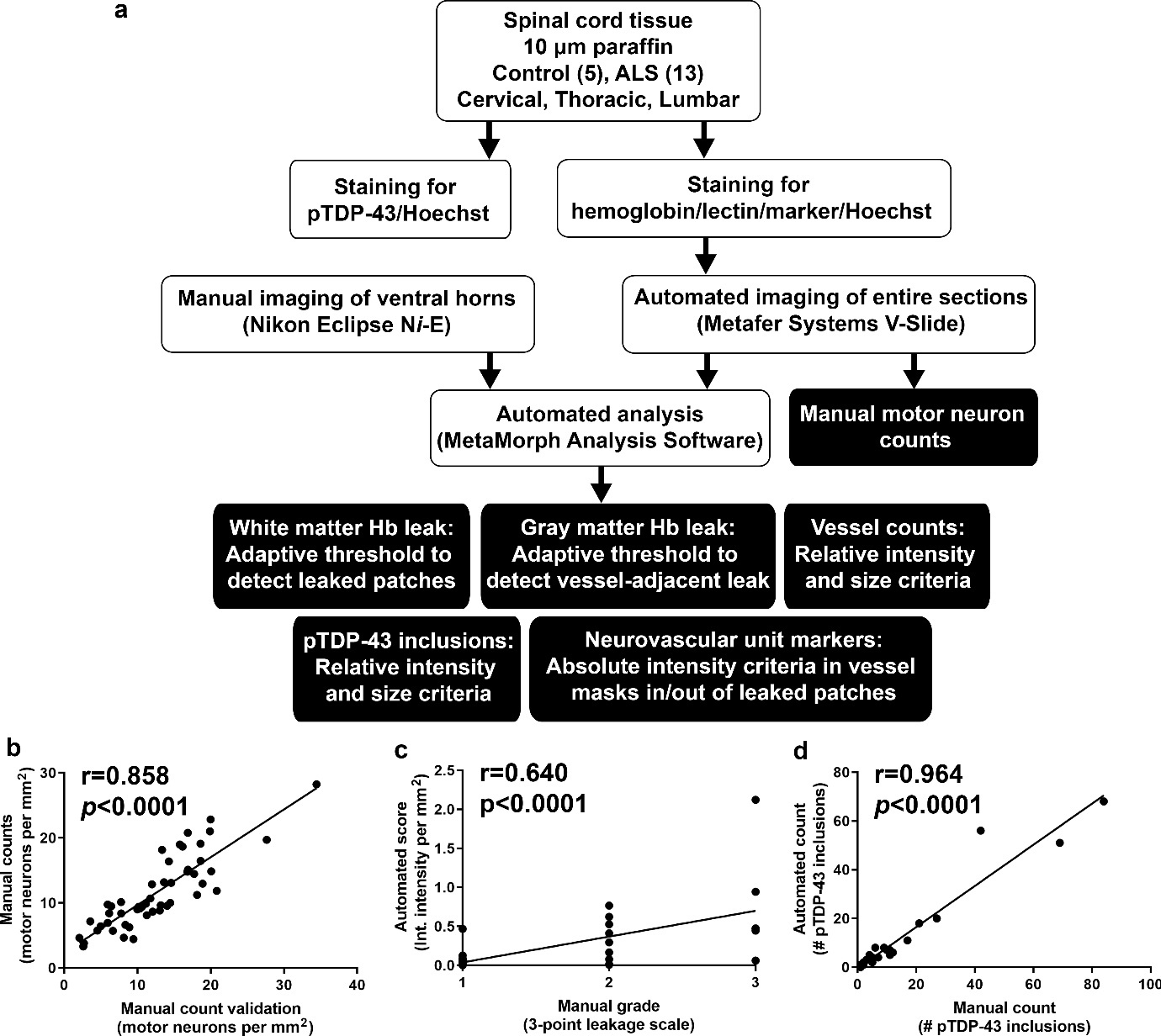
**

**Supplementary Figure 3. Imaging and quantification workflow**

(**a**) Workflow of tissue staining and quantitative image analysis methodology where 10 µm sections of paraffin-embedded human spinal cord were stained for either pTDP-43 or for hemoglobin and lectin with a marker of interest. Subsequent imaging with the VSlide or Nikon, and manual counting analysis for SMI-32-positive motor neurons, or automated analysis for pTDP-43 pathology, hemoglobin leakage, neurovascular unit markers, and vessel density. (**b**) Pearson correlation of manual motor neuron counting against manual counting of motor neurons performed by a blinded observer. (**c**) Pearson correlation of automated pTDP-43 inclusion analysis against manual counting of pTDP-43 inclusions. (**d**) Pearson correlation of automated hemoglobin analysis against manual scoring by a blinded scorer on a semi-quantitative 3-point leakage scoring scale.

**
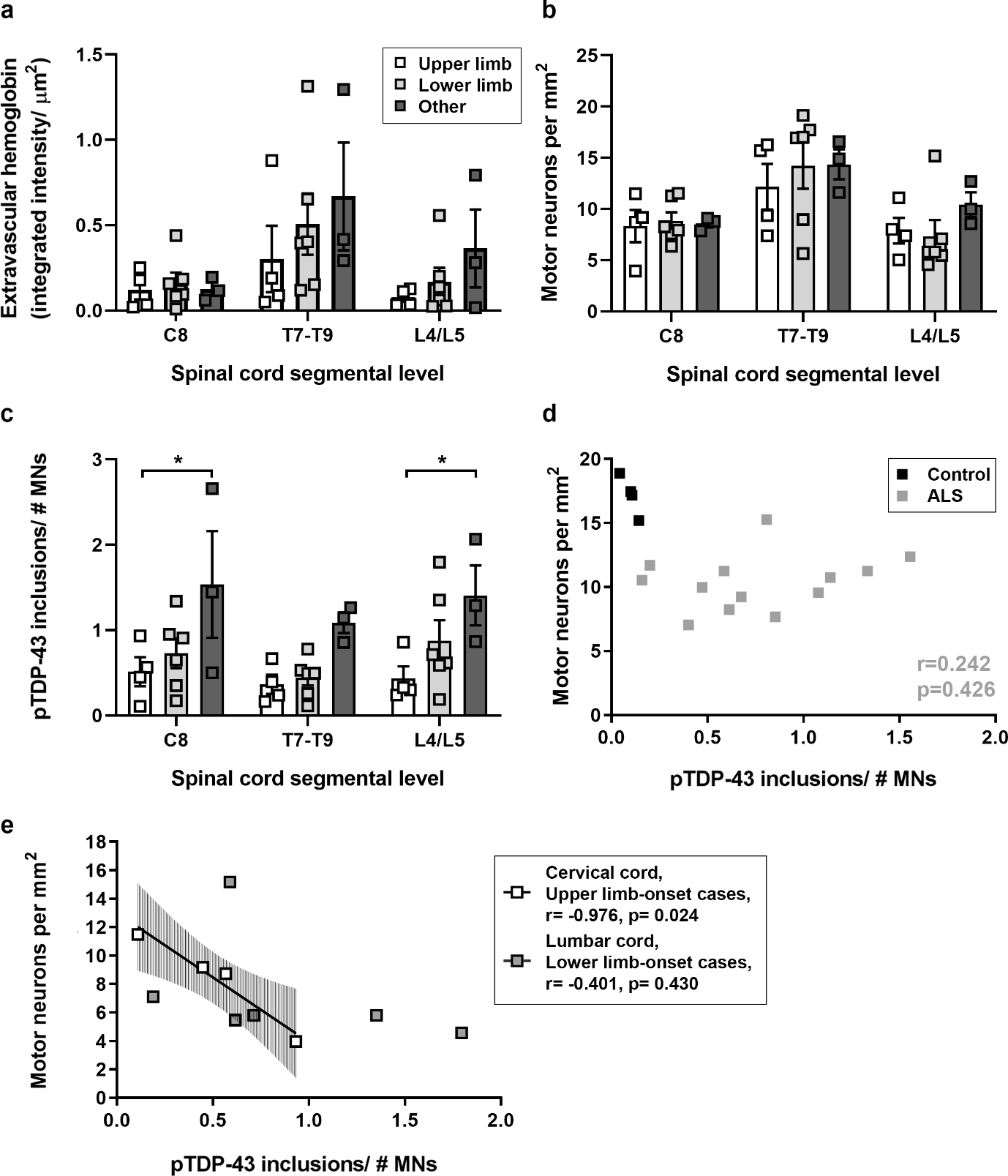
**

**Supplementary Figure 4. Patterning of neither extravascular hemoglobin nor motor neuron pathology were affected by site of ALS onset; pTDP-43 inclusion load predicted motor neuron loss only in cervical cord of upper-limb onset cases**

(**a**) Quantification of extravascular hemoglobin (both grey and white matter) at individual segmental levels C8, T7-T9 and L4/L5 with respect to site of ALS symptom onset. (**b**) Motor neuron numbers per area of ventral horn at individual segmental levels C8, T7-T9, and L4/L5 with respect to site of ALS symptom onset; upper limb, lower limb, or ‘other’ (respiratory, bulbar, frontotemporal dementia). (**c**) Phospho-TDP-43 inclusions/ number of motor neurons at individual segmental levels C8, T7-T9, and L4/L5 with respect to site of ALS symptom onset. C8 and L4/L5: Upper limb vs. other. Data shown as mean ± SD (n = 3-6 cases per group), with statistical significance determined using two-way ANOVA with Tukey’s post-test; *p ≤ 0.05. (**d**) Scatter plot of motor neuron numbers per area of ventral horn against phospho-TDP-43 inclusions/ number of motor neurons, averaged across three levels of spinal cord. Pearson correlation conducted on ALS cases only (ns). (**e**) Scatter plot of motor neuron numbers per area of ventral horn against phospho-TDP-43 inclusions/ number of motor neurons, at the level of the cord corresponding to onset. Pearson correlations for cervical cord in upper-limb onset cases, and lumbar cord in lower-limb onset cases.

**
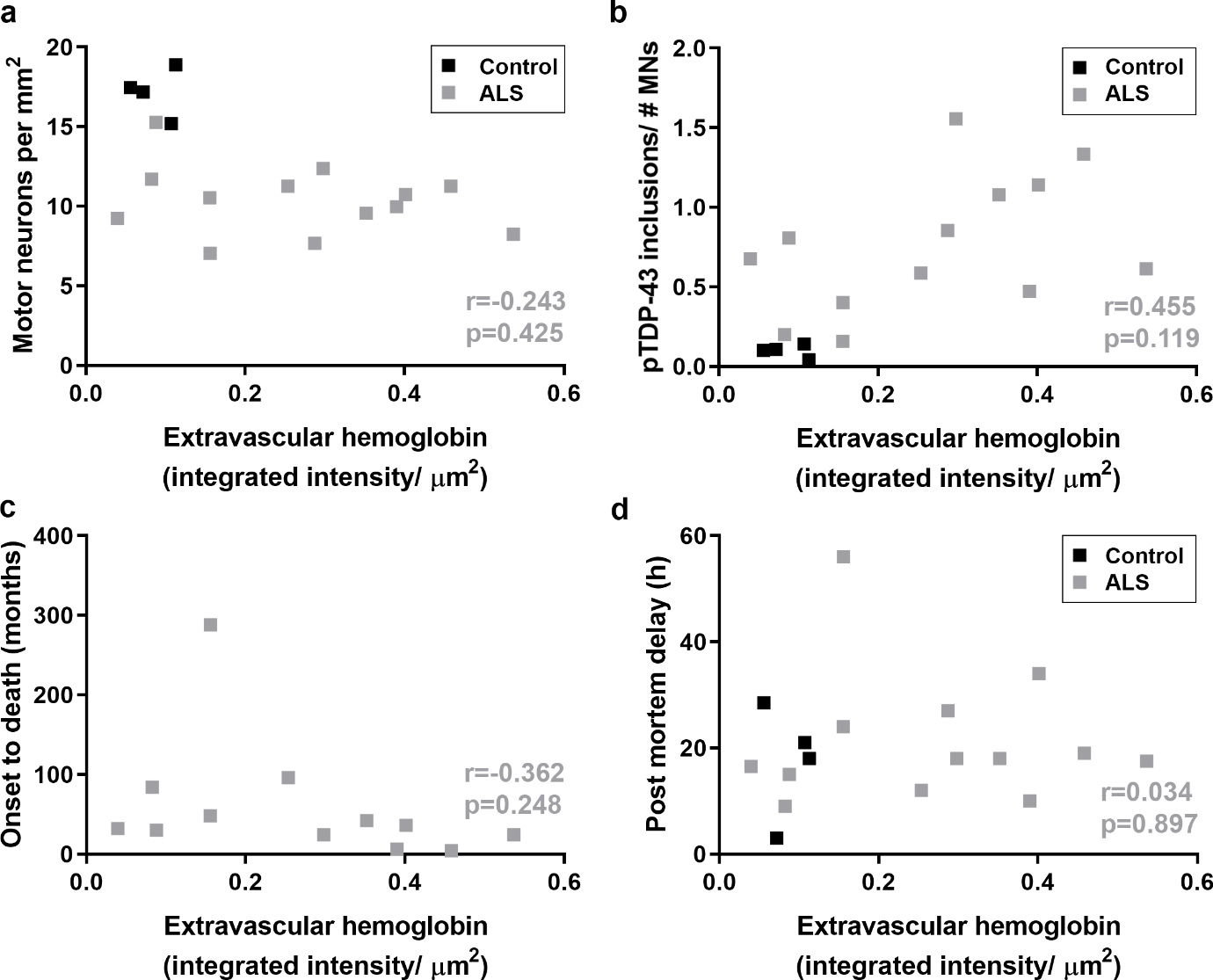
**

**Supplementary Figure 5. Extravascular hemoglobin did not correlate with motor neuron pathology, disease, or spinal cord tissue collection delay**

Scatter plots of extravascular hemoglobin (both grey and white matter averaged across cervical C8, thoracic T7-T9, and lumbar L4/L5 levels) in ALS spinal cord against motor neuron numbers per area of ventral horn (**a**), phospho-TDP-43 inclusions/ number of motor neurons (**b**), disease duration (**c**), and post mortem delay (**d**). Pearson correlation conducted on ALS cases only, all ns.

**Supplementary Table 1. Review of blood-brain and blood-spinal cord barrier disruption in human ALS studies**

**Summary of findings**

| **Change** | **Component** | **Marker/ process** | **Change in ALS versus control** | **Comments** |
| --- | --- | --- | --- | --- |
| **NVU changes** | Endothelia | Degeneration | ↑ [2, 3] | Rare or unquantified |
|  |  | Microvascular density | ↑ [2, 4] |  |
|  |  | TDP-43 proteinopathy | ↑ [5] | Very rare |
|  | Tight junctions | Degeneration | ↔ [3] |  |
|  |  | Claudin-5 | ↔ [6]  ↓ [2] |  |
|  |  | ZO-1 | ↓ [2, 6] |  |
|  |  | Occludin | ↓ [2, 6, 7] |  |
|  |  | JAM-1 | ↓ [2] |  |
|  | Adherens junctions | VE-cadherin | ↓ [2] |  |
|  | Efflux pumps | P-gp | ↑ [8] |  |
|  |  | BCRP | ↑ [8] |  |
|  | Pericytes | Degeneration | ↑ [2-4] | Ventral horn |
|  |  | PDGFRβ | ↓ [9] |  |
|  |  | αSMA | ↓ [4] |  |
|  | Basement membrane | Collagen deposition | ↓ [7, 10]  ↑ [2, 3] |  |
|  | Astrocyte endfeet | Detachment | ↑[7] |  |
|  |  | TDP-43 proteinopathy | ↔ [11]  ↑[5] | Very rare |
| **BSCB changes** | Integrity | IgG leakage | ↑ [2, 9, 12, 13] |  |
|  |  | Hemoglobin leakage | ↑ [9] |  |
|  |  | C3 complement leakage | ↑ [12] |  |
|  |  | Albumin leakage | ↔ [12, 13] |  |
|  |  | Fibrin leakage | ↑ [9] |  |
|  |  | Thrombin leakage | ↑ [9] |  |
|  |  | Microbleeds | ↔ [14]  ↑ [9] |  |

NVU, neurovascular unit; BSCB, blood-spinal cord barrier; ZO-1, zona occludins-1; JAM-1, junctional adhesion molecule-1; VE-cadherin, vascular endothelial-cadherin; P-gp, P-glycoprotein; BCRP, breast cancer resistance protein; PDGFRβ, platelet-derived growth factor receptor beta; αSMA, alpha smooth muscle actin; IgG, immunoglobulin G.

**Detailed findings**

| **Reference** | **Number of cases** | **Regions** | **Method** | **Markers (target)** | **Quantification** | **Main findings** |
| --- | --- | --- | --- | --- | --- | --- |
| **[2]** | NNDC (n= 18)  ALS (n= 25) | Medulla, and cervical and lumbar spinal cord, anterior horn gray matter, and, pyramidal tract or lateral funiculi white matter | Electron microscopy | NA | Qualitative | Medulla: Ultrastructure of capillaries in ALS varied compared with NDCC, including but not limited to signs of pericyte degeneration, multiple endothelial cell layers, edema, lipofuscin inclusions, large accumulations of disorganised collagen.  Spinal cord gray matter: Ultrastructure of capillaries in ALS varied compared with NDCC, including swollen endothelial cells, complete pericyte degeneration, and large accumulations of collagen.  Spinal cord white matter: Ultrastructure of capillaries in ALS varied compared with NDCC, where most capillaries displayed a thin layer of endothelial cells. |
|  |  |  |  |  | Quantitative (microvascular collagen expansion) | 2-2.5-times increase in basement membrane collagen accumulation in microvessels in ALS white and gray matter compared with NDCC.  Higher basement membrane collagen accumulation was observed in cervical white matter, relative to medulla or lumbar in ALS. |
|  |  |  | Chromogenic labelling | Sirius red (collagen) | Quantitative (microvascular density) | 2-fold increase in microvascular density in the ALS lumbar spinal cord, relative to NDCC.  No significant difference was observed in cervical spinal cord or medulla. |
|  |  |  | Fluorescent IHC | CD31 (endothelia)  CD105 (endothelia)  Immunoglobulin (BSCB leakage) | Qualitative | Discontinuous expression of CD31 and CD105 observed along endothelial lining observed in ALS tissues, relative to NNDC.  Extravascular immunoglobulin observed in ALS tissues, but not in NNDC. |
|  |  |  | Western blot | Occludin (tight junction)  Claudin-5 (tight junction)  JAM-1 (tight junction)  ZO-1 (tight junction)  VE-cadherin (adherens junction) | Quantitative (band densities relative to GAPDH) | Significant decreases in **most** tight junction and adhesions proteins in gray and white matter in ALS spinal cord and medulla, relative to NDCC.   - **ZO-1:** decreased in medulla gray matter, and cervical and lumbar spinal cord gray and white matter - **Occludin:** decreased in the medulla and cervical spinal cord gray and white matter - **Claudin-5:** decrease in medulla gray and white matter, and cervical spinal cord gray matter - **JAM-1:** decreased in cervical and lumbar spinal cord gray matter - **VE-cadherin:** decreased in medulla and cervical spinal cord white matter |
| **[3]** | NNDC (n= 12)  ALS (n= 12) | Lumbar spinal cord anterior horn gray matter | Electron microscopy | NA | Quantitative (manual count of basement membrane replication, or endothelial or pericyte degenerated nuclei or vacuolated cytoplasm, per capillary) | Tight junctions were well-preserved in ALS, even in endothelia with degenerated nucleus. Greater proportion of endothelia (ALS, 1.6%; Control, 0.8%) and pericytes (ALS, 1.6%; Control, 1.1%) with a degenerated nucleus in ALS cases than controls, but still extremely rare.  Greater proportion of endothelia (ALS, 3.6%; Control, 1.1%) and pericytes (ALS, 3.8%; Control, 1.1%) with a vacuolated cytoplasm in ALS cases than controls, but still rare.  Greater proportion of vessels with basement membrane replication (ALS, 8.2%; Control, 3.4%) in ALS cases than controls. |
|  |  |  |  |  | Semi-quantitative (manual count of vessels with collagen fibre content classed as mild, moderate, or severe) | Greater proportion of vessels with moderate or severe perivascular collagen fibre deposition (ALS, 30.1%; Control, 18.7%) in ALS cases than controls. |
| **[4]** | NNDC (n= 6)  ALS (n= 25) | Lumbar spinal cord anterior and dorsal horn gray matter | Chromogenic IHC | CD34 (endothelia)  Alpha-smooth muscle actin (pericytes- also labels vascular smooth muscle cells) | Quantitative (counts of microvessels (<10 µm) in anterior and dorsal horns, entire hemisection)  Quantitative (counts of vessels co-labelled for CD34 and α-SMA) | Increased microvascular density in ALS anterior horn compared to controls, particularly in cases that received artificial respiratory support.  Increased microvascular density in ALS dorsal horn compared to controls, only in cases that received artificial respiratory support.  Decreased pericyte coverage of microvessels in ALS anterior horn compared to controls. |
| **[5]** | NNDC (not given)  ALS (n= 14)  FTLD-TDP (n= 7) | Spinal cord anterior horn  Frontal cortex | Chromogenic IHC | TDP-43 (pan TDP-43)  pTDP-43 S403/404 (proteinopathy)  pTDP-43 S409/410 (proteinopathy)  GFAP (astrocytes)  CD146 (endothelia) | Qualitative | Rare TDP-43 proteinopathy in spinal cord blood vessel walls (n= 7/14 ALS, 50%) in ALS cases but not controls. This included rare TDP-43 nuclear clearing from vascular nuclei in anterior horn and white matter; and rare pTDP-43 deposits in vascular cells or perivascular spaces. |
|  |  |  | Fluorescent IHC | pTDP-43 S403/404 (proteinopathy)  GFAP (astrocytes)  CD68 (macrophages) | Qualitative | Rare TDP-43 proteinopathy in close proximity to GFAP-positive astrocytes. |
| **[6]** | NNDC (n= 16)  ALS (n= 34) | Lumbar spinal cord | qRT-PCR | ZO-1 (tight junction)  Occludin (tight junction)  Claudin 5 (tight junction)  Beta-actin (housekeeping gene) | Quantitative | **ZO-1 mRNA expression:**  Reduced ~30% in sALS lumbar spinal cord compared with NNDC.  Appeared reduced in fALS lumbar spinal cord but not significant compared with NNDC.  Reduced ~40% in lumbar spinal cord in all ALS cases ≤57 y compared with NNDC.  **Occludin mRNA expression:**  Appeared reduced in sALS lumbar spinal cord but not significant compared with NNDC.  Reduced ~55% in fALS lumbar spinal cord compared with NNDC.  Reduced ~40% in lumbar spinal cord in all ALS cases ≤57 y compared with NNDC.  Positive correlation between age at death from ALS (but not NNDC) and occludin mRNA expression.  **Claudin 5 mRNA expression:**  Not significantly different between ALS and NNDC. |
| **[7]** | NNDC (n= 3)  ALS (n= 3) | Spinal cord anterior horn gray matter, level not given | Fluorescent IHC | CD31 (endothelia)  GFAP (astrocytes)  Collagen IV (basement membrane)  Occludin (tight junction) | Qualitative | Astrocyte endfeet staining for GFAP retained and enriched around blood vessels but not directly opposed to them in ALS-‘detachment’ of astrocytic endfeet from endothelia in ALS cases.  Decrease in collagen IV- or occludin-positive vascular structures in ALS cases. |
| **[8]** | NNDC (n= 2)  ALS (n= 3) | Lumbar spinal cord | Western blot | P-glycoprotein (endothelial efflux pump)  Breast cancer resistance protein (BCRP; endothelial efflux pump)  GAPDH (housekeeping protein) | Qualitative | Increased P-gp and BCRP protein expression in ALS compared to controls. |
| **[9]** | NNDC (n= 5)  sALS (n= 8)  fALS (n= 3) | Cervical spinal cord anterior horn gray matter | Chromogenic labelling | Podocalyxin (capillaries)  Hemosiderin (BSCB leakage) | Qualitative | Prussian blue-positive hemosiderin deposits predominantly found surrounding podocalyxin-positive capillaries. |
|  |  |  |  |  | Quantitative  (manual counts of perivascular Prussian-blue positive hemodesirin deposits) | 2.5-fold increase in Prussian blue-positive hemosiderin deposits per mm^2^.  Extent of perivascular hemosiderosis significantly positively correlated with extravascular hemoglobin integrated density. |
|  |  |  | Fluorescent IHC | PDGFRβ (pericytes- also labels vascular smooth muscle cells)  Hemoglobin (BSCB leakage)  Glycophorin A (CD235a; erthyrocytes)  Fibrinogen (BSCB leakage)  Thrombin (BSCB leakage)  NeuN (neurons)  UEA-1 lectin (endothelia)  Immunoglobulin G (IgG; BSCB leakage) | Quantitative  (automated measurements of immunopositive area, integrated intensity, and immunopositive cell number) | 3.1-fold increase in extravascular hemoglobin integrated density in ALS relative to NNDC, with no difference between sALS and fALS.  19% reduction in PDGFRβ-positive pericyte coverage and 54% reduction in PDGFRβ-positive pericyte number in ALS versus NNDC.  Extent of extravascular hemoglobin integrated density significantly negatively correlated with PDGFRβ-positive pericyte coverage (combining sALS, fALS, and NNDC). |
|  |  |  |  |  | Qualitative | Extravascular CD235a-positive erythrocytes observed in ALS but not NNDC.  Extravascular IgG co-labelled with extravascular hemoglobin in ALS and NNDC.  Fibrin- and thrombin-positive accumulations, including within NeuN-positive neurons) observed ALS but not NNDC. |
| **[10]** | NNDC (n=21)  ALS (n= 10) | Cervical spinal cord anterior horn gray matter, posterior funiculus, lateral funiculus | Chromogenic IHC | Masson trichrome stain (vessel walls) | Quantitative (digital analysis of blood vessel wall and total field areas) | No signiﬁcant difference in vessel wall area between ALS and controls. |
|  |  |  | Electron microscopy | NA | Qualitative | Fragmented and disorganised perivascular collagen bundles in cervical spinal cord anterior horn and posterior lateral funiculus in ALS but not controls. |
|  |  |  | Liquid chromato-graphy | Collagen (basement membrane) | Quantitative (collagen content) | Reduced collagen content in ALS compared to controls. |
| **[11]** | NNDC (n= 17)  ALS (n= 82) | Lumbar spinal cord  Motor cortex | Fluorescent IHC | pTDP-43 S403/404 (proteinopathy)  GFAP (astrocytes) | Qualitative | No astrocytic perivascular pTDP-43 detected. |
| **[12]** | NNDC (n= 4)  ONDC (n= 8)  ALS (n= 16) | Cervical and lumbar spinal cord, motor cortex | Fluorescent IHC | IgG (BSCB leakage)  C3 complement (BSCB leakage)  Albumin (BSCB leakage) | Semi-quantitative (5-point scale of IgG or complement deposition) | Deposition of IgG / C3 complement immune complexes in spinal cord anterior horn gray matter (n= 6/16 ALS, 37.5%) and in motor cortex (n= 6/16 ALS, 38.5%) in ALS compared to controls. Immune complexes were associated with astrocytes and oligodendrocytes.  No deposition of albumin. |
| **[13]** | NNDC (n= 10)  ALS (n= 15) | Cervical and lumbar spinal cord, motor cortex, cerebellum | Chromogenic IHC | IgG (BSCB leakage)  Albumin (BSCB leakage)  HLA-DR (macrophage activation)  Myelin basic protein (oligodendrocytes) | Quantitative (manual counting of IgG-positive motor neurons) | Deposition of IgG in spinal cord motor neurons (n= 13/15 ALS, 86.8%) and in motor cortex pyramidal neurons (n= 6/11 ALS, 54.6.5%) in ALS compared to controls.  Deposition of IgG in spinal cord anterior horn and corticospinal tract macrophages (n= 12/15 ALS, 80%) in ALS compared to controls.  Deposition of IgG in astrocytes was not specific to ALS.  No deposition of albumin. |
| **[14]** | NNDC (n= 12)  ALS (n= 12) | Whole brain | 7T T2*-weighted MRI imaging | Hypointensities due to cerebral microbleeds | Qualitative | No evidence for cerebral microbleeds in ALS. |

**References**

1. Frostell, A., et al., *A Review of the Segmental Diameter of the Healthy Human Spinal Cord.* Front Neurol, 2016. **7**: p. 238.

2. Garbuzova-Davis, S., et al., *Impaired blood-brain/spinal cord barrier in ALS patients.* Brain Res, 2012. **1469**: p. 114-28.

3. Sasaki, S., *Alterations of the blood-spinal cord barrier in sporadic amyotrophic lateral sclerosis.* Neuropathology, 2015. **35**(6): p. 518-28.

4. Yamadera, M., et al., *Microvascular disturbance with decreased pericyte coverage is prominent in the ventral horn of patients with amyotrophic lateral sclerosis.* Amyotroph Lateral Scler Frontotemporal Degener, 2015. **16**(5-6): p. 393-401.

5. Ferrer, I., et al., *TDP-43 Vasculopathy in the Spinal Cord in Sporadic Amyotrophic Lateral Sclerosis (sALS) and Frontal Cortex in sALS/FTLD-TDP.* J Neuropathol Exp Neurol, 2021.

6. Henkel, J.S., et al., *Decreased mRNA expression of tight junction proteins in lumbar spinal cords of patients with ALS.* Neurology, 2009. **72**(18): p. 1614-6.

7. Miyazaki, K., et al., *Disruption of neurovascular unit prior to motor neuron degeneration in amyotrophic lateral sclerosis.* J Neurosci Res, 2011. **89**(5): p. 718-28.

8. Jablonski, M.R., et al., *Selective increase of two ABC drug efflux transporters at the blood-spinal cord barrier suggests induced pharmacoresistance in ALS.* Neurobiol Dis, 2012. **47**(2): p. 194-200.

9. Winkler, E.A., et al., *Blood-spinal cord barrier breakdown and pericyte reductions in amyotrophic lateral sclerosis.* Acta Neuropathol, 2013. **125**(1): p. 111-20.

10. Ono, S., et al., *Collagen abnormalities in the spinal cord from patients with amyotrophic lateral sclerosis.* J Neurol Sci, 1998. **160**(2): p. 140-7.

11. Nolan, M., et al., *Quantitative patterns of motor cortex proteinopathy across ALS genotypes.* Acta Neuropathol Commun, 2020. **8**(1): p. 98.

12. Donnenfeld, H., R.J. Kascsak, and H. Bartfeld, *Deposits of IgG and C3 in the spinal cord and motor cortex of ALS patients.* J Neuroimmunol, 1984. **6**(1): p. 51-7.

13. Engelhardt, J.I. and S.H. Appel, *IgG reactivity in the spinal cord and motor cortex in amyotrophic lateral sclerosis.* Arch Neurol, 1990. **47**(11): p. 1210-6.

14. Verstraete, E., et al., *No evidence of microbleeds in ALS patients at 7 Tesla MRI.* Amyotroph Lateral Scler, 2010. **11**(6): p. 555-7.
